# Light-induced LLPS of the CRY2/SPA1/FIO1 complex regulating mRNA methylation and chlorophyll homeostasis in *Arabidopsis*

**DOI:** 10.1038/s41477-023-01580-0

**Published:** 2023-12-08

**Authors:** Bochen Jiang, Zhenhui Zhong, Lianfeng Gu, Xueyang Zhang, Jiangbo Wei, Chang Ye, Guifang Lin, Gaoping Qu, Xian Xiang, Chenjin Wen, Maureen Hummel, Julia Bailey-Serres, Qin Wang, Chuan He, Xu Wang, Chentao Lin

**Affiliations:** 1 Basic Forestry and Plant Proteomics Research Center, Fujian Agriculture and Forestry University, Fuzhou, China; 2 Department of Molecular Cell and Developmental Biology, University of California, Los Angeles, CA USA; 3 Department of Chemistry, The University of Chicago, Chicago, IL USA; 4 Center for Plant Cell Biology and Department of Botany and Plant Sciences, University of California, Riverside, CA USA; 5 Shandong Laboratory of Advanced Agricultural Sciences at Weifang, Peking University Institute of Advanced Agricultural Sciences, Weifang, China

**Keywords:** Cell signalling, Light responses

## Abstract

Light regulates chlorophyll homeostasis and photosynthesis via various molecular mechanisms in plants. The light regulation of transcription and protein stability of nuclear-encoded chloroplast proteins have been extensively studied, but how light regulation of mRNA metabolism affects abundance of nuclear-encoded chloroplast proteins and chlorophyll homeostasis remains poorly understood. Here we show that the blue light receptor cryptochrome 2 (CRY2) and the METTL16-type m^6^A writer FIONA1 (FIO1) regulate chlorophyll homeostasis in response to blue light. In contrast to the CRY2-mediated photo-condensation of the mRNA adenosine methylase (MTA), photoexcited CRY2 co-condenses FIO1 only in the presence of the CRY2-signalling protein SUPPRESSOR of PHYTOCHROME A (SPA1). CRY2 and SPA1 synergistically or additively activate the RNA methyltransferase activity of FIO1 in vitro, whereas CRY2 and FIO1, but not MTA, are required for the light-induced methylation and translation of the mRNAs encoding multiple chlorophyll homeostasis regulators in vivo. Our study demonstrates that the light-induced liquid–liquid phase separation of the photoreceptor/writer complexes is commonly involved in the regulation of photoresponsive changes of mRNA methylation, whereas the different photo-condensation mechanisms of the CRY/FIO1 and CRY/MTA complexes explain, at least partially, the writer-specific functions in plant photomorphogenesis.

## Abstract (web summary)

*Arabidopsis* photoreceptor CRY2 controls chlorophyll homeostasis by blue light-induced liquid–liquid phase separation of the CRY2/SPA1/FIO1 complex, resulting in activation of the m^6^A writer FIO1, regulating mRNAs encoding chlorophyll homeostasis proteins.

## Main

Cryptochromes (CRYs) are blue light receptors that mediate light regulation of photomorphogenesis and photosynthesis in plants^1,2^. *Arabidopsis* has two CRY photoreceptors, CRY1 and CRY2, that control various aspects of photoresponses in a partially redundant manner. CRYs interact with transcriptional regulators and the E3 ubiquitin ligases to regulate transcription, protein degradation, chloroplast protein homeostasis and photosynthesis^1,2^. Light is also known to control translation^3–5^, but the mechanism underlying light regulation of translation is not clear. *Arabidopsis* CRY2 is a nuclear photoreceptor that undergoes light-induced homo-oligomerization and liquid–liquid phase separation (LLPS) to become physiologically active^6–9^. CRYs physically interact with many CRY-signalling proteins to regulate protein expression^1,2^. For example, CRY2 interacts with SUPPRESSOR of PHYTOCHROME A (SPA1), which is a subunit of the E3 ubiquitin ligase CUL4^COP1/SPA1^, to inhibit CUL4^COP1/SPA1^-dependent polyubiquitination and proteolysis of various light-signalling proteins^10–14^. SPA1 interacts with CRY2 in a blue light-dependent manner, and it is required for all major physiological activities of CRY2 (refs. ^1,2^). SPA1 is a WD-domain protein that belongs to a small gene family of four related genes, *SPA1* to *SPA4*, which have partially redundant functions in photomorphogenesis^13^. On the other hand, mRNA adenosine methylase (MTA) interacts with CRY2 in a light-independent manner, but it is rapidly co-condensed to the CRY2 photobody in response to blue light^15^. It was hypothesized that the light-induced LLPS of CRY2 co-condenses MTA to increase its local concentration in the CRY2 photobody, leading to increased methylation and stability of mRNA and light control of the circadian clock in plants^15^.

*N*^6^-methyladenosine (m^6^A) is the most abundant internal modification of eukaryotic mRNAs that regulates mRNA splicing, nuclear export, degradation and translation^16–19^. It has been reported that mRNA methylation is important for protecting photosynthesis from photodamage^20^, but the underlying mechanism is unknown. The m^6^A RNA methylation is catalysed by two evolutionarily conserved eukaryotic RNA methyltransferases, the METTL3/METTL14-type and the METTL16-type m^6^A writers. Human METTL3/METTL14 contains two catalytic subunits, methyltransferase-like 3 (METTL3) and methyltransferase-like 14 (METTL14)^21–23,24–26^. METTL3/METTL14 deposits m^6^A to the A residues of the RRACH (R = A/G, H = A/C/U) or related motifs in many mRNAs^18,27,28^. In contrast, the metazoan METTL16-type writers are single-subunit methyltransferases that methylate limited RNA substrates with the preference of hairpin structure and different sequence contexts, such as U6 snRNA or MAT2A preRNA^29–33^. Both types of m^6^A writer are evolutionarily conserved in plants. *Arabidopsis* MTA/MTB and FIO1 are the counterparts of the human METTL3/METTL14 and METTL16, respectively^34,35,36^. *Arabidopsis* MTA is required for embryogenesis^35^, photomorphogenesis^15,37^ and stress responses^38,39^, whereas FIO1 is required for maintaining the appropriate period length of the circadian clock, flowering time and photomorphogenesis^36,40–43^. In contrast to the metazoan METTL16, *Arabidopsis* FIO1 deposits m^6^A marks to not only U6 snRNA but also thousands of mRNA substrates^40–43^. Plant FIO1 appears to have the substrate specificity more similar to that of the metazoan METTL3 than that of the metazoan METTL16 (refs. ^32,33,40–43^).

Blue light positively regulates mRNA methylation in *Arabidopsis*, and CRYs are known to mediate blue-light stimulation of mRNA methylation by the photo-condensation of the MTA writer complex^15,37^. However, it is not fully understood exactly how CRY2 photo-condensation affects mRNA methylation or whether CRYs also regulate the MTLL16-type m^6^A writer FIO1. In this Article, we investigated these questions. We found that the *Arabidopsis* mutant *fio1-1*, but not *mta*, exhibits a low-chlorophyll phenotype similar to that of the *cry1cry2* mutant, indicating a distinct function of FIO1 from that of MTA. Based on a multiple omics clustering analysis, we identified six chlorophyll homeostasis regulator (*CHR*) genes encoding CHRs that undergo CRY/FIO1-activated mRNA methylation and translation to maintain the chlorophyll homeostasis in response to light. In contrast to MTA, photoexcited CRY2 alone does not co-condense FIO1, and the light-dependent CRY2-interacting protein SPA1 acts as a nuclear chaperone that facilitates co-condensation of FIO1 to the CRY2 photobody. Importantly, the CRY C-terminal extension (CCE) domain of CRY2 and the WD domain of SPA1 synergistically or additively activate the m^6^A methyltransferase activity of FIO1 in vitro. The *spa123* and *spa134* triple mutants impaired in multiple *SPA* genes also showed decreases in chlorophyll and photoresponsive mRNA methylation and translation of the *CHR* genes. These results support a mechanistic model that explains an epitranscriptomic mechanism regulating chlorophyll homeostasis in response to light. According to this model, photoexcited CRY2 interacts with SPA1 to co-condense FIO1, forming the CRY2/SPA1/FIO1 trimolecular condensates. CRYs and SPA1 synergistically activate FIO1 within the CRY2/SPA1/FIO1 condensates, facilitating deposition of m^6^A markers and increased translation of the mRNAs that encode chloroplast proteins required for maintaining chlorophyll homeostasis and photosynthesis in response to light.

## Results

### CRYs and FIO1 are required for chlorophyll homeostasis

**Fig. 1:**
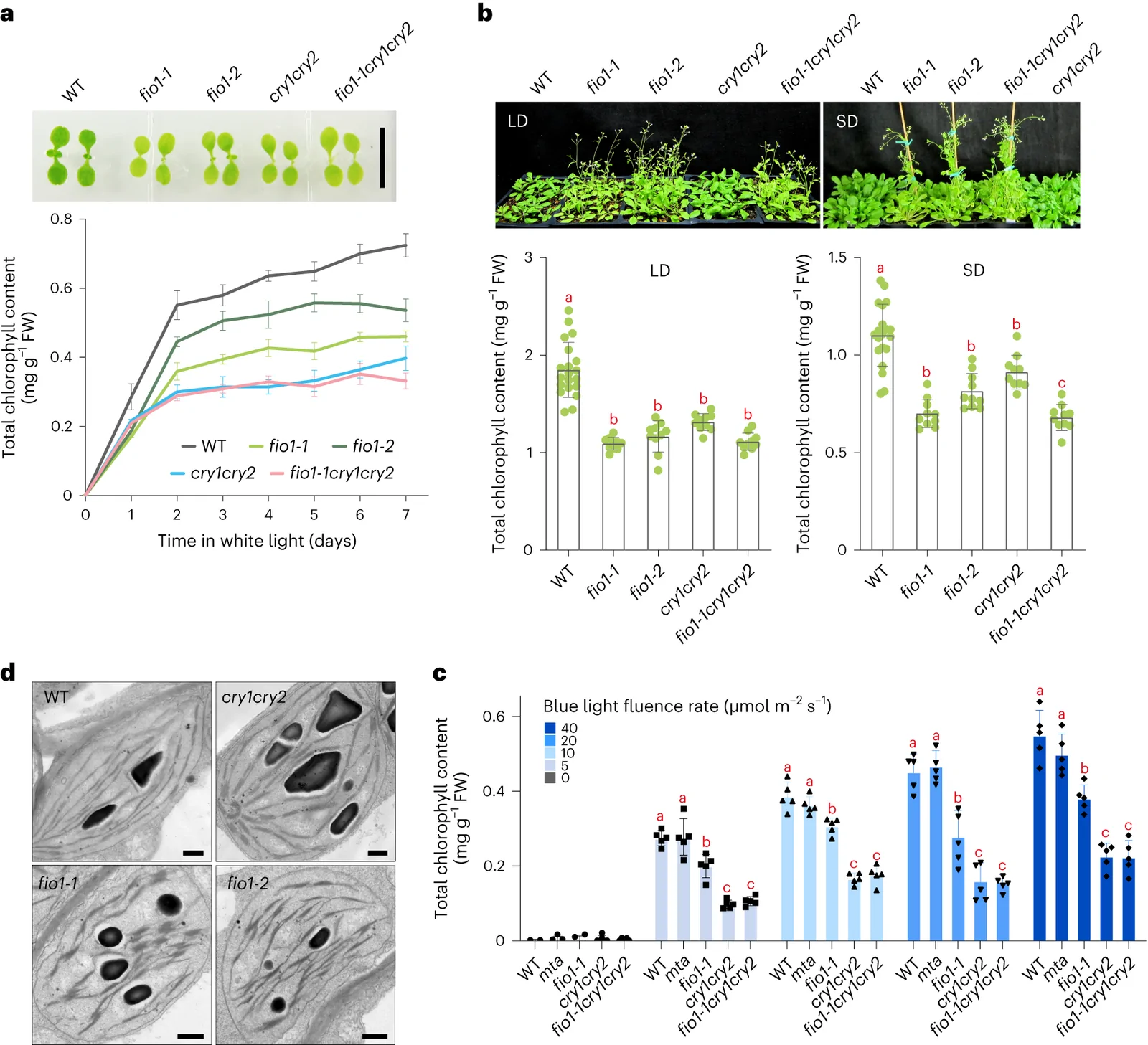
CRYs and FIO1 are required for maintaining normal chlorophyll homeostasis. **a**, Phenotypes of WT (Col) and mutants grown under white light for 6 days (top) and total chlorophyll content (bottom; chlorophyll *a* + *b*, mg g^−1^ fresh weight (FW)) of different genotypes grown under white light at the indicated days after germination (mean ± s.d., *n* = 4, 3, 3, 3, 2 average values from independent experiments). Scale bar, 10 mm. **b**, Phenotypes (top) and total chlorophyll content (bottom) of 4-week-old plants grown in long day (LD) photoperiods or 6-week-old plants grown in short days (SD) (mean ± s.d., *n* = 20, 10, 10, 10, 10 independent experiments). The lowercase letters indicate statistically significant differences (*P* < 0.05) by one-way ANOVA test followed by Tukey’s multiple comparisons test. **c**, Total chlorophyll content of 6-day-old WT and mutant seedlings grown under blue light with indicated fluence rates (mean ± s.d., *n* = 5 independent experiments). The lowercase letters indicate statistically significant differences (*P* < 0.05) by one-way ANOVA test followed by Tukey’s multiple comparisons test. The exact *P* values (**b** and **c**) are provided in Supplementary Table 16. **d**, Transmission electron micrographs of chloroplasts of WT and mutant seedlings grown in blue light (25 μmol m^−2^ s^−1^) for 6 days. Scale bar, 5 μm. Three independent experiments show similar results. Source data

**Extended Data Fig. 1:**
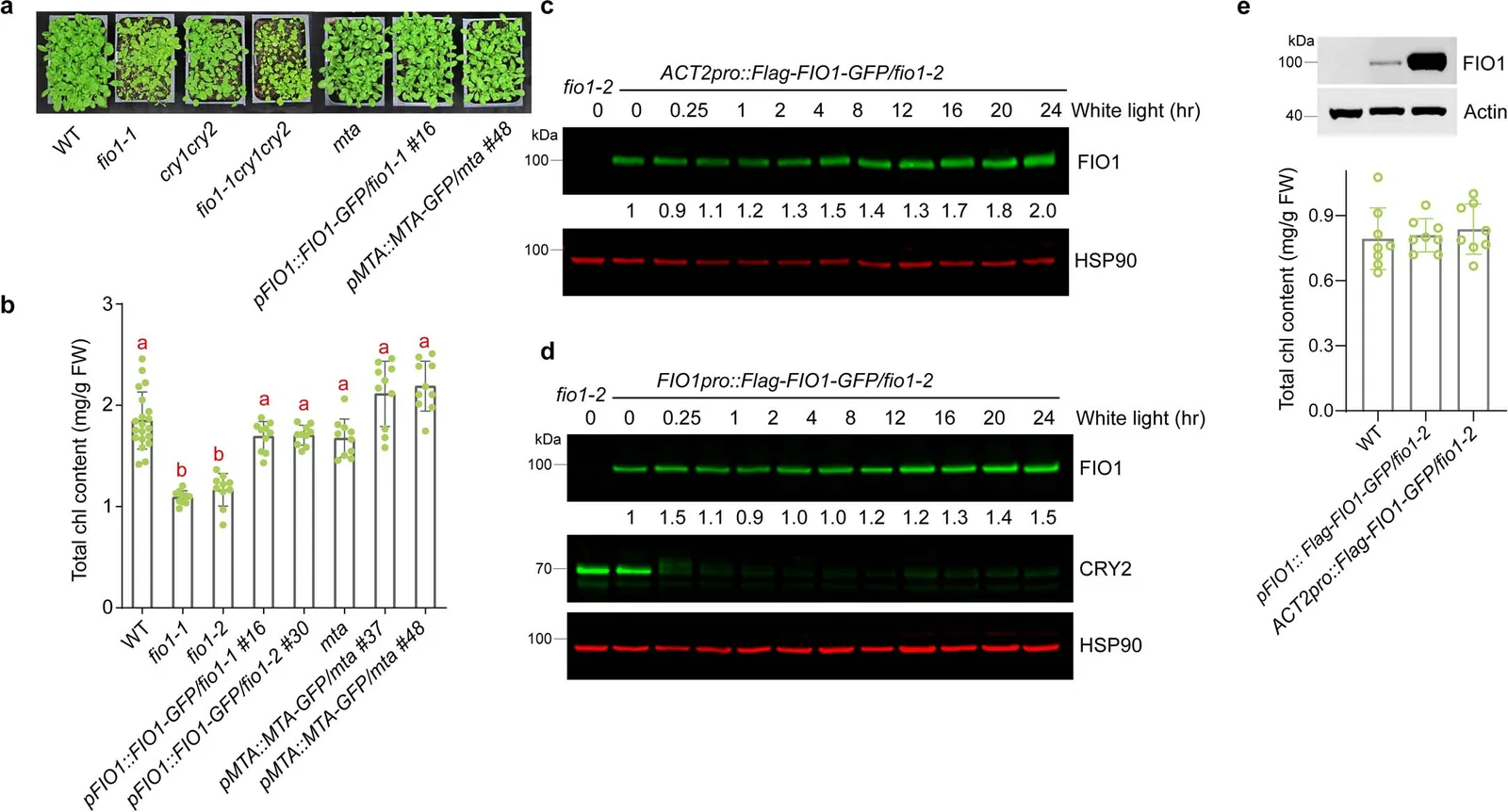
The FIO1 protein is not apparently regulated by light. **a**. Phenotypes of different genotypes grown in long-day conditions for 4 weeks. **b**. Total chlorophyll content of 4-week-old plants grown in long day photoperiods. The lowercase captions of different letters indicate statistically significant differences (p < 0.05) by one way ANOVA test followed by Tukey’s multiple comparisons test (mean ± s.d., n = 20,10,10,10,10,10,10,10 independent experiments). **c-d**. Immunoblot showing the FIO1 protein levels in transgenic plants expressing *pACT2:: Flag-FIO1-GFP/fio1-2* (**c**) or *pFIO1:: Flag-FIO1-GFP/fio1-2* (**d**). 6-day-old etiolated seedlings were exposed to white light for the indicated time before harvest. Immunoblotting was performed to detect FIO1 proteins with anti-Flag antibody. HPS90 detected by the anti-HSP90 antibody was served as the loading control. CRY2 detected by anti-CRY2 antibody was used to validate the light response of seedlings. *fio1-2* seedlings were used as the negative control. The immunoblot results were quantified using ImageJ software. FIO1 protein levels were normalized to the HSP90 loading control. The relative protein levels were shown below the blots, with the protein level at 0 h set as 1.0. The exact *P* values are provided in Supplementary Table 16. **e**. Upper, immunoblot showing the FIO1 protein levels in transgenic plants expressing *pACT2:: Flag-FIO1-GFP/fio1-2* or *pFIO1:: Flag-FIO1-GFP/fio1-2*. 6-day-old seedlings were grown under blue light (25 μmol m^−2^ s^−1^) before harvest. Immunoblotting was performed to detect FIO1 proteins with anti-Flag antibody. Actin detected by the anti-HSP90 antibody served as the loading control. Lower, total chlorophyll content of plants grown under blue light (25 μmol m^−2^ s^−1^) (mean ± s.d., n = 8 independent experiments). Source data

The *FIO1* gene was originally identified in a mutant screen, and the loss-of-function *fio1* mutant exhibits abnormal period lengths of the circadian clock and accelerated floral initiation^36^. We noticed that the *fio1* mutant plants exhibited a pale green phenotype (Fig. 1a,b). Quantitative analyses demonstrate that the total chlorophyll content is lower in seedlings of both *fio1-1* and *fio1-2* mutant alleles at various developmental stages or grown under different white or blue light conditions (Fig. 1a–c). The low-chlorophyll phenotype of the *fio1* mutants represents abnormal chlorophyll homeostasis, which may result from decreased synthesis or increased breakdown of chlorophyll. This is consistent with the recent report that the *fio1* mutants showed lower quantum efficiency of PSII reaction centres^43^. Interestingly, this low-chlorophyll phenotype was not observed in the *mta* mutant (Fig. 1c and Extended Data Fig. 1a,b), suggesting that FIO1 and MTA play distinct roles in maintaining the appropriate chlorophyll homeostasis in *Arabidopsis*. No additive or synergistic low-chlorophyll phenotype was observed in the *fio1-1cry1cry2* triple mutant (Fig. 1a–c). The low-chlorophyll phenotype of *fio1-1cry1cry2* triple mutant resembles that of the *cry1cry2* mutant. This observation indicates that the CRY photoreceptors may regulate chlorophyll homeostasis by multiple mechanisms, including regulation of the activity of FIO1. The low-chlorophyll phenotype of the *cry1cry2* and *fio1* mutants does not seem to affect gross morphology of chloroplasts (Fig. 1d). The level of *FIO1* mRNA expression appears unchanged in response to blue light (Supplementary Table 1), but levels of the FIO1 protein increase modestly in response to light (Extended Data Fig. 1c,d). Transgenic lines overexpressing *FIO1* show no abnormal chlorophyll contents (Extended Data Fig. 1e), indicating that FIO1 is regulated by light but the level of the FIO1 protein may not be rate-limiting in maintaining chlorophyll homeostasis. We hypothesize that the light- and CRY/FIO1-dependent but MTA-independent mRNA methylation is required to maintain the normal protein expression and chlorophyll homeostasis in light-grown plants.

### Photoresponsive of CRYs, MTA and FIO1 across multiple omics

**Fig. 2:**
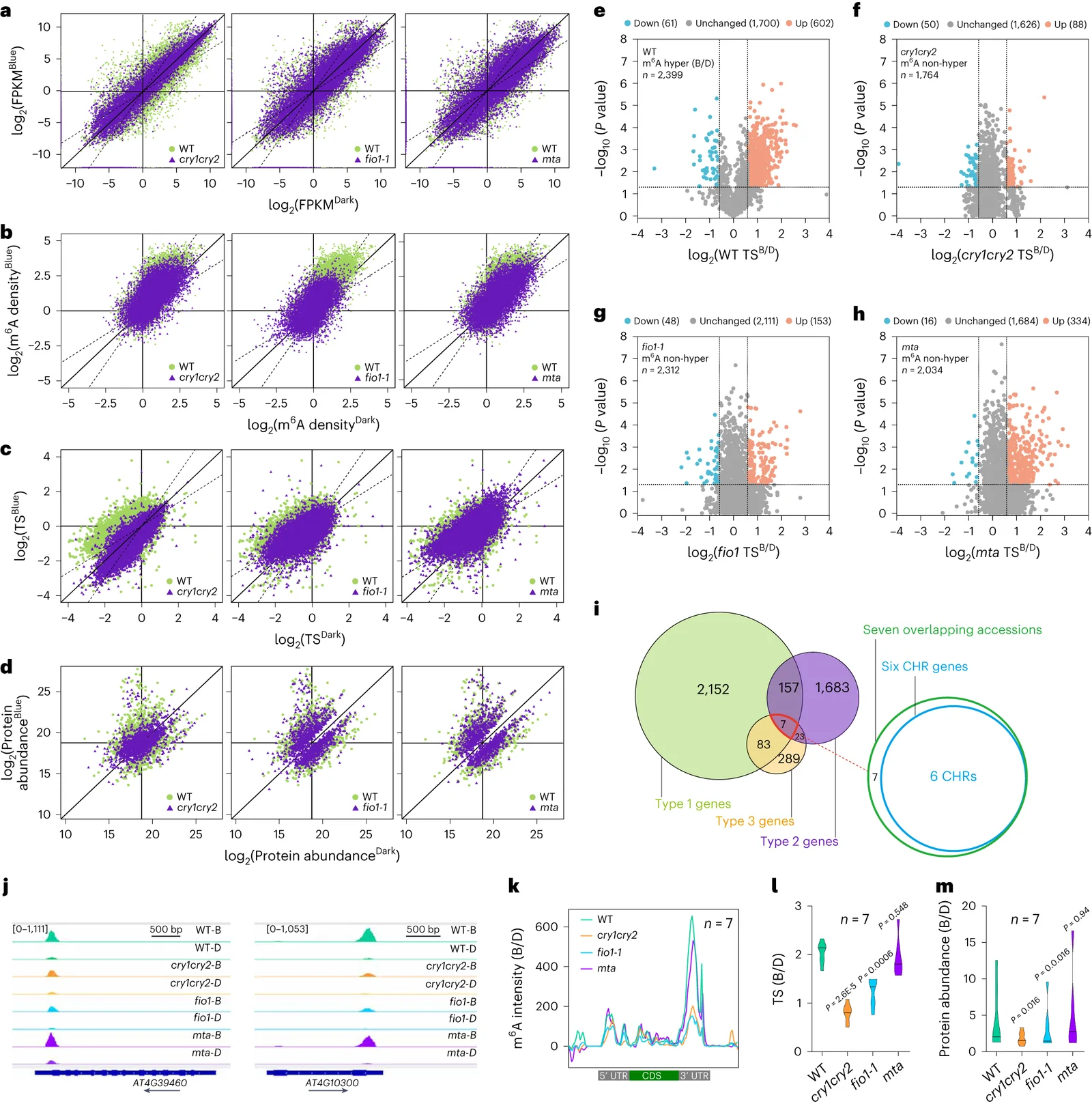
CRYs and FIO1 mediate blue light-induced mRNA methylation and translation of the *CHR* genes. **a**–**c**, Scatter plots showing the photoresponsive transcriptomic (**a**), epitranscriptomic (**b**) and translation state (TS) (**c**) changes. The dashed lines indicate >1.5× photoresponsive changes in mRNA abundance. **d**, Scatter plots showing the photoresponsive proteomic changes of the eight-sample cohort. The differentially expressed accessions of the WT (B/D > 1.5, *P* < 0.01, two-tailed Student’s *t*-test) were first selected and plotted, and those accessions in the mutants are plotted for comparison. In **a**–**d**: WT, green dot; three mutants, purple dot. Seedlings were grown in dark (abscissa) or blue light (ordinate) (25 μmol m^−2^ s^−1^) for 6 days before collection. **e**–**h**, The volcano plots showing photoresponsive changes of translation states (WT, TS^B/D^) associated with the light-induced mRNA methylation in the wild-type plants (**e**) and in the *cry1cry2* (**f**), *fio1-1* (**g**) and *mta* (**h**) mutants. ‘WT m^6^A hyper (B/D)’ is defined by m^6^A-B/D^WT^ >1.5. ‘m^6^A non-hyper’ in the mutant indicated is defined by m^6^A-B/D^WT^ >1.5 and m^6^A-B/D^mt^ <1.5, mt: mutants. B/D: blue/dark. |TS^B/D^| >1.5, *P* < 0.05, two-tailed Student’s *t*-test. **i**, Venn graph shows overlaps of the type 1, type 2 and type 3 genes. Six of the seven overlapping genes encode CHRs. **j**, Genomic visualization of m^6^A density maps of representative CRY/FIO1-dependent photo-activation of mRNA methylation genes. **k**, The distribution of photoresponsive mRNA m^6^A intensity of the seven genes shown in **i** mapped along relative mRNA position in different genotypes. **l**,**m**, Violin plots comparing the photoresponsive changes of translation state (**l**) and protein abundance (**m**) of the seven accessions shown in **i**. The ratio of translation state (**l**) or protein abundance (**m**) between seedlings grown in blue light and darkness is shown for the indicated genotypes. *P* values are determined by two-tailed Student’s *t*-test (**l**) or the two-sided Wilcoxon test (**m**). Source data

**Extended Data Fig. 2:**
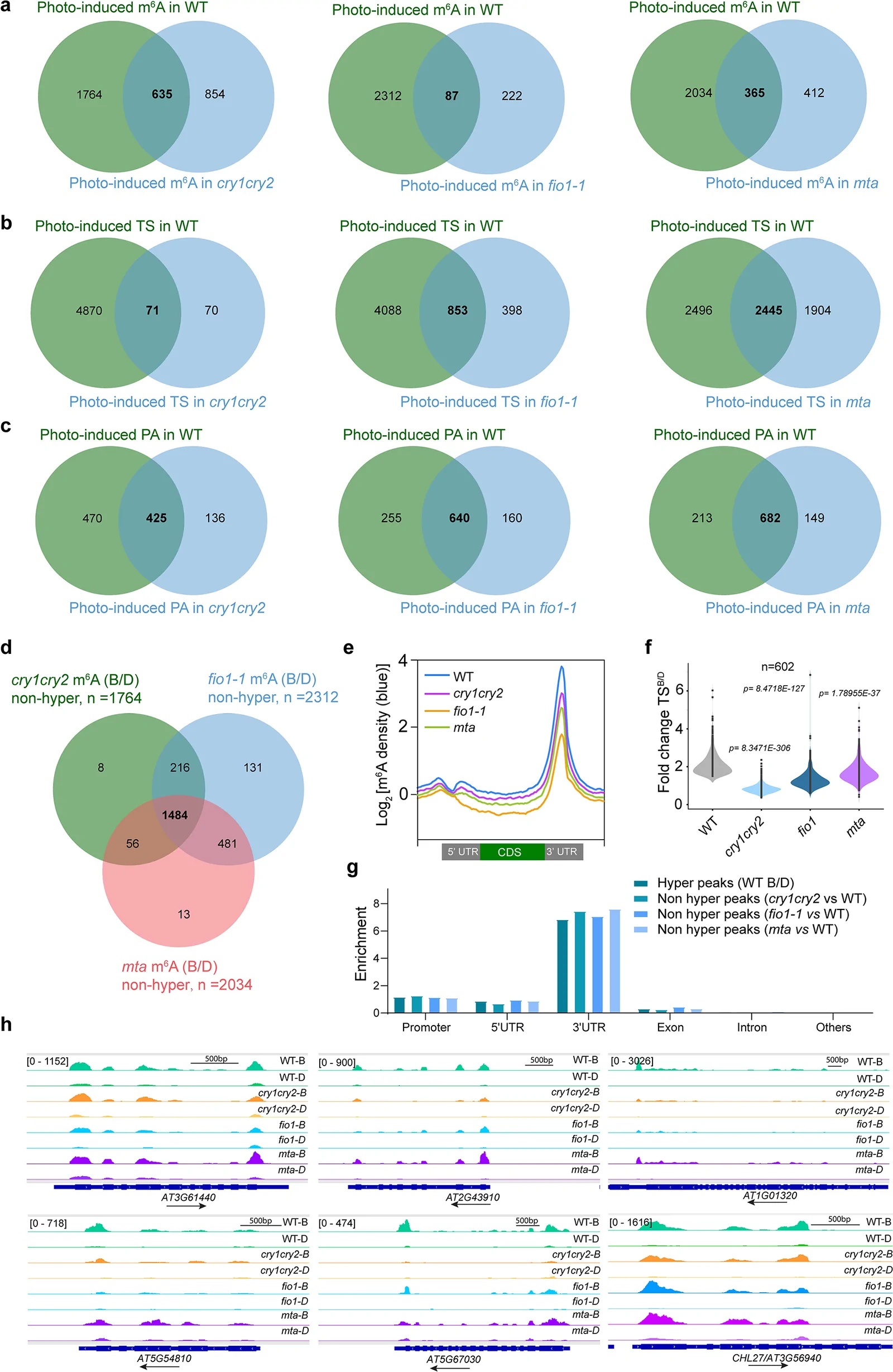
CRYs and m^6^A writers regulate photoresponsive changes of the transcriptome, epitranscriptome, translatome, and proteome. **a-c**. The overlapping genes showing significant photoresponsive genes (FC^B/D^ > 1.5, *P* value < 0.05) between WT and each mutant in epitranscriptome (**a**), translatome (**b**) and proteome (**c**). **d**. The Venn graph shows overlaps of photoresponsive m^6^A epitranscriptome of the indicated genotypes. The ‘m^6^A non-hyper’ are defined by m^6^A-B/D^WT^ > 1.5 & m^6^A-B/D^mt^ < 1.5, WT: wild-type, mt: mutants. **e**. The distribution of m^6^A intensity (in blue light conditions) mapped along relative mRNA position in different genotypes. **f**. Violin plots comparing the photoresponsive changes of translation state of the 602 genes shown in Fig. 2e. The ratio of Translation state between seedlings grown in blue light (Blue, B) and darkness (Dark, D) are shown for the indicated genotypes, *P* values are calculated by two-tailed Student’s *t*-test. **g**. The enrichment of the m^6^A peaks at different positions across the mRNA transcript in photo-induced m^6^A peaks (hyper peaks) and non-hyper peaks in mutants. The enrichment was calculated by the number of m^6^A peaks normalized by the length of the region. **h**. Genomic visualization of m^6^A density maps of individual transcripts whose m^6^A peaks are CRY/FIO1-dependent photo-induced and the control gene *CHL27*. Source data

**Extended Data Fig. 3:**
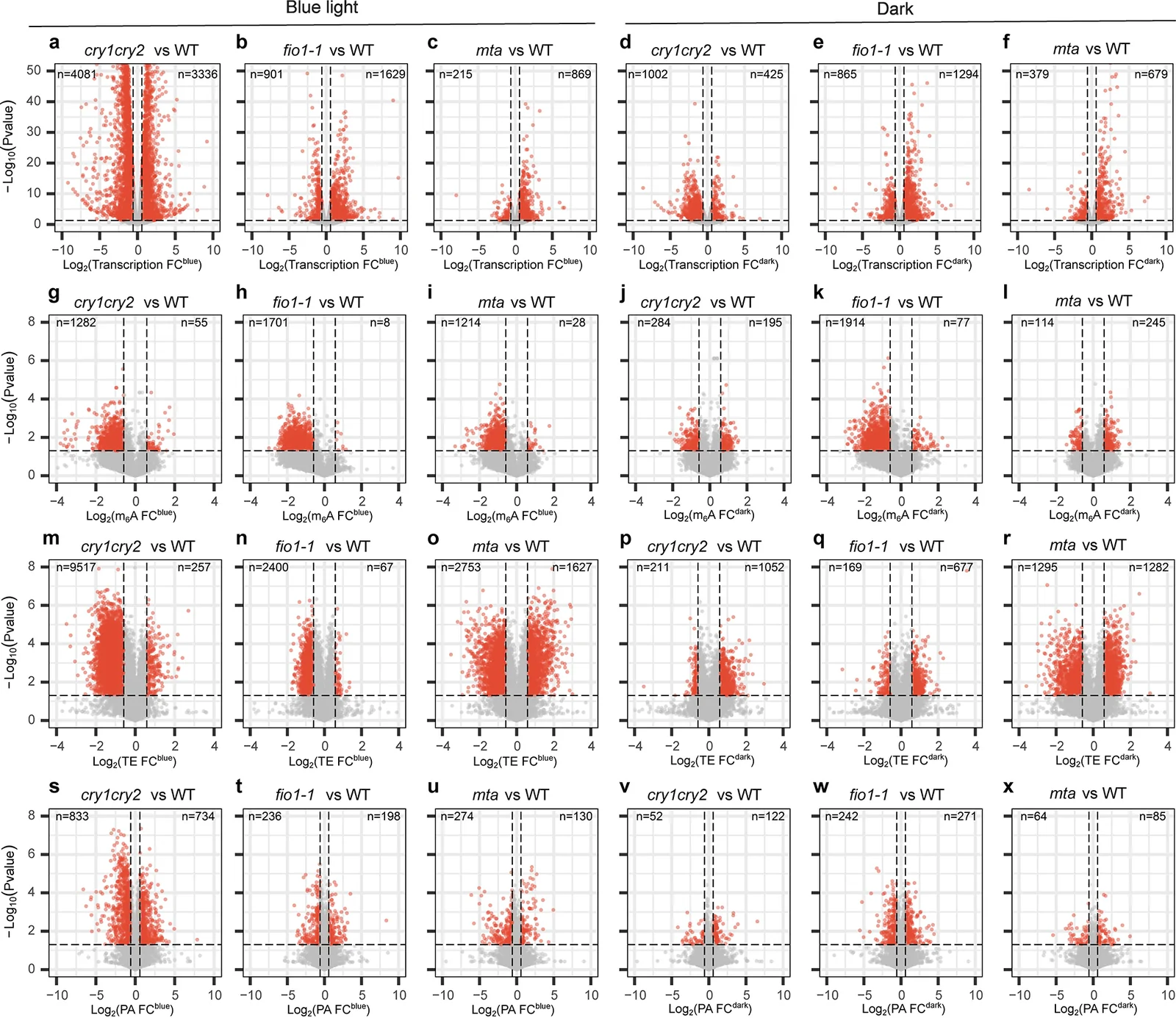
Volcano plots showing the omics changes of the indicated mutants. The number of genes (or m^6^A peaks) that show changes in the indicated mutants (*cry1cry2*, *fio1-1*, and *mta*) in comparison to the wild-type (WT) in transcriptome (**a-f**), m^6^A epitranscriptome (**g-l**), translatome (**m-r**), and proteome (**s-x**). The R package DEseq2 was used to analyze the differential expression of transcriptomes. The avo function in R was used to calculate the fold changes and *P* values for the m^6^A epitranscriptome, translatome, and proteome. The red-colored dots indicate genes (or m^6^A peaks) that show significant changes (FC < 1/1.5 or >1.5, *P* < 0.05, two-tailed Student’s *t*-test) in the indicated mutants. The numbers of significantly down- and up-regulated genes/m^6^A peaks are included in the top left and top right in each plot, respectively. Source data

To investigate this hypothesis, we analysed the transcriptomes, m^6^A epitranscriptomes, translatomes and proteomes derived from 6-day-old seedlings of four genotypes (wild type or WT, *cry1cry2*, *fio1-1* and *mta* mutants) grown in continuous darkness (D) or blue light (B). For simplicity, we refer to these eight samples as the ‘8-sample cohort’ in this report. We used the previously established methods in this experiment, including the MeRIP method for epitranscriptome profiling^15,44^, the translating ribosome affinity purification (TRAP) method for translatome analyses^45,46^ and the label-free quantitative mass spectrometry (MS) methods for proteome analyses^47^. We plotted binary logarithms of the datasets of transcriptomes (Fig. 2a), m^6^A epitranscriptome (Fig. 2b), translatome (Fig. 2c) and proteome (Fig. 2d) derived from individual genotypes grown in the dark (abscissa) or blue light (ordinate) and compared overall photoresponsive changes of each mutant with that of the WT (Fig. 2a–d, purple versus green). The datapoints located further away from the diagonal line in each plot represent mRNA that exhibits stronger photoresponsive changes of the steady-state mRNA abundance (Fig. 2a), m^6^A methylation of mRNA (Fig. 2b), translation state (Fig. 2c) or protein abundance (Fig. 2d), respectively. We define the photoresponsiveness of individual gene expression product in different datasets by the fold change (FC) between samples of the light-grown and dark-grown seedlings (B/D > 1.5, *P* < 0.05 or false discovery rate (FDR) <0.05), and use these parameters to further analyse our omics datasets (Fig. 2, Extended Data Figs. 2 and 3 and Supplementary Tables 1–10). Figure 2a shows that, in comparison with that of the WT, the distribution of transcriptomic datapoints of the *cry1cry2* mutant shrank toward the diagonal line, indicating an overall decline of the photoresponsiveness of mRNA expression in the *cry1cry2* mutant. However, no similar change is found in transcriptomic datasets of the *fio1-1* and *mta* mutants (Fig. 2a). This and additional analysis of the transcriptomic datasets (Extended Data Fig. 3a–f and Supplementary Table 1) demonstrate that the CRY photoreceptors, but not the two m^6^A writers, determine the photoresponsive changes of steady-state mRNA abundance.

Figure 2b shows the photoresponsive change of m^6^A density, or changes of m^6^A deposition per unit length of RNA, in response to light. The datapoints representing blue light-dependent changes of mRNA methylation in the WT plants apparently concentrate more above the diagonal line of quadrant I (Fig. 2b, green), demonstrating the light-induced increase of m^6^A mRNA methylation in the WT. This is consistent with that reported previously^15^. There is a global downshifted distribution of the datapoints derived from all three mutants (Fig. 2b, purple, Extended Data Figs. 2a and 3g–l and Supplementary Tables 2–6), indicating the diminished light induction of mRNA methylation in all three mutants. For example, in the WT seedlings, 2,399 mRNA accessions showed blue light-induced m^6^A deposition or photoresponsive hyper-methylation (B/D > 1.5, *P* < 0.05 or FDR <0.05). Of those 2,399 mRNA accessions, only about 26% (635/2,399), 3.6% (87/2,399) or 15% (365/2,399) showed the blue light-induced hyper-methylation in the *cry1cry2*, *fio1-1* and *mta* mutants, respectively (Extended Data Fig. 2a and Supplementary Tables 3–6), suggesting that all three genes are required for the blue-light stimulation of mRNA methylation. Importantly, the *cry1cry2*, *fio1-1* and *mta* mutants shared 61% (1484/2399) ‘non hyper’ mRNA accessions that lost photoresponsive hyper-methylation (Extended Data Fig. 2d), demonstrating the overlapping substrate specificity of MTA and FIO1 catalysing the photoresponsive m^6^A methylation, and that CRYs mediate blue-light activation of both MTA- and FIO1-dependent mRNA methylation. Consistent with that reported previously^15,39–41,43,48^, the 3′ untranslated region (UTR) of mRNAs is methylated more than other regions of transcripts. Consistently, 3′ UTR of mRNAs also showed more pronounced photoresponsive hyper-methylation for mRNAs in the WT plants and loss of this photoresponse in mRNAs of all three mutants (Extended Data Fig. 2e,g).

Although the decreased mRNA methylation in the *fio1-1* and *mta* mutants may alter stability of mRNAs, such changes may be masked in the steady-state transcriptomes by various feedback regulatory mechanisms controlling the photoresponsive transcription. This may explain the apparent change of steady-state transcriptome in the *cry1cry2* mutant and the lack of similar changes in the *fio1-1* and *mta* mutations (Fig. 2a). Because changes in mRNA methylation may also affect translation of the respective mRNAs, we analysed photoresponsive changes of ribosome-associated mRNAs to examine their translation states for the 8-sample cohort. Figure 2c shows that the datapoints representing photoresponsive changes in translation state of the WT seedlings tend to distribute above the diagonal line of quadrants I and III (Fig. 2c, green), indicating a blue light induction of mRNA recruitment to ribosomes or a blue light-induced translation. All three mutants showed diminished light induction of translation, but the effect on photoresponsive translation can be ranked in the order of *cry1cry2* > *fio1-1* > *mta* (Fig. 2c, purple). For example, of the 4,941 mRNA accessions that exhibited blue light-induced translation state in the WT plants, about 1.4% (71/4941), 17% (853/4941) or 49% (2,445/4,941) showed light-induced translation in the *cry1cry2*, *fio1-1* or *mta* mutant, respectively (Extended Data Figs. 2b and 3m–r and Supplementary Table 8). The writer-specific m^6^A deposition on the same or different mRNA substrates may explain why the *mta* mutant suffers the least with respect to the blue light-induced stimulation of translation. We tested this proposition by two analyses. First, we analysed the light-induced, hyper-methylated and hyper-translated mRNA accessions in the WT plants and the three mutants. Of the 2,399 mRNA accessions that showed photoresponsive hyper-methylation in the WT plants, 25% (602/2399) showed light-induced hyper-translation in the WT plants. But the photoresponsive translation of these 602 mRNAs diminished significantly in the three mutants, especially in *cry1cry2* and *fio1-1* mutants (Extended Data Fig. 2f). Second, we analysed in more detail how loss of photoresponsive hyper-methylation of mRNA affected light-induced translation in the three mutants (Fig. 2e–h). Of the 2,399 photoresponsive hyper-methylated mRNA accessions identified in the WT, 73% (1764/2,399), 96% (2,312/2,399) or 84% (2,034/2,399) lost the photoresponsive hyper-methylation to become ‘m^6^A non-hyper’ mRNAs in *cry1cry2*, *fio1-1* or *mta* mutant, respectively (Fig. 2f–h, *cry1cry2* non-hyper, *fio1-1* non-hyper, *mta* non-hyper). In contrast to 25% (602/2,399) hyper-methylated mRNA accessions that showed light-induced translation in the WT plants, only about 5% (88/1,764), 7% (153/2,312) or 17% (334/2,034) of the ‘m^6^A non-hyper’ mRNA accessions showed light-induced translation in the *cry1cry2*, *fio1-1* or *mta* mutant, respectively (Fig. 2e–h). In other words, the mRNA accessions that no longer undergo light-induced hyper-methylation are much less likely to show light-induced translation, especially in the *cry1cry2* and *fio1-1* mutants. The observation that the *cry1cry2* and *fio1-1* mutants suffered more pronounced impairment in the light-induced translation than that of the *mta* mutant would be explained, at least partially, by the writer-specific mRNA methylation.

We next analysed the proteomes of the 8-sample cohort. In comparison with transcriptomes, epitranscriptomes and translatomes, the proteomes appear to exhibit generally weaker photoresponsive changes (Fig. 2d, Extended Data Figs. 2c and 3s–x and Supplementary Table 9). For example, in comparison with thousands of mRNA that showed significant changes (B/D > 1.5, *P* < 0.05 or FDR <0.05) in transcriptome, epitranscriptome and translatome, we detected 895, 561, 800 or 831 proteins that showed the significant increase (B/D > 1.5, *P* < 0.05) of protein abundance in the WT, *cry1cry2*, *fio1-1* or *mta* mutant, respectively (Extended Data Fig. 2c). This result may be explained by two possibilities: the relatively low sensitivity of mass-spectrometry analyses of proteins and/or relatively more feedback regulatory steps involved in controlling the steady-state levels of protein abundance. Among the 895 proteins that showed light-dependent increase of protein abundance in the WT plants, about 47% (425/895), 72% (640/895) or 76% (682/895) proteins continue to show the light-dependent increase of protein abundance in the *cry1cry2*, *fio1-1* or *mta* mutant, respectively (Extended Data Fig. 2c), implying that the *cry1cry2* mutant suffers more pronounced defects in the photoresponsive steady-state proteomes than the *fio1-1* or *mta* mutant. This observation would be explained by the fact that CRYs, but not the m^6^A writers, are known to regulate photoresponsive transcription and protein turnover.

*Arabidopsis* genome encodes at least 58 chlorophyll synthesis enzyme (CSE) proteins that are directly involved in chlorophyll biosynthesis (Supplementary Table 12 and databases referenced within). Given that light promotes chlorophyll synthesis in *Arabidopsis* and that CRYs are multifunctional gene-expression regulators but MTA and FIO1 are presently known only for their activity regulating mRNA methylation, stability or translation, any of those 58 *CSE* genes that show light-induced increase of mRNA expression, methylation and protein abundance in the CRYs/FIO1-dependent but MTA-independent manner may explain the genotype-specific low-chlorophyll phenotype observed (Fig. 1). However, we did not find any *CSE* genes that satisfy these criteria (Supplementary Tables 12 and 13 and Extended Data Figs. 2 and 3). For example, although the levels of mRNAs of *HEMA1* that encodes glutamyl-tRNA reductase GluTR and *GENOMES UNCOUPLED 4* (*GUN4*) that encodes the porphyrin-binding activator of magnesium chelatase are significantly lower in the light-grown *cry1cry2* and *fio1-1* mutants than those of the WT and *mta* mutant, we failed to detect the corresponding changes in the levels of protein abundance that may explain the genotype-specific low-chlorophyll phenotype (Supplementary Tables 12 and 13). Similarly, other *CSE* genes that showed the CRY-dependent photoinduction of mRNA and protein expression also failed to show the FIO1-dependent but MTA-independent photoinduction of mRNA and protein expression or mRNA methylation, translation and protein expression (Supplementary Tables 12 and 13 and Extended Data Figs. 2 and 3). To explain the genotype-specific low-chlorophyll phenotype (Fig. 1), we proposed an alternative hypothesis that CRYs and FIO1, but not MTA, may promote methylation and translation of mRNAs encoding non-CSE proteins, referred to as CHRs, that are required to maintain the normal chlorophyll homeostasis in light-grown plants.

### Identification of CRY/FIO1-dependent CHRs

To test the alternative CHR hypothesis described above, we used a four-step multi-omics clustering approach to identify the genes that showed the RNA methylation, translation and protein expression patterns that may explain the genotype-specific low-chlorophyll phenotype (Fig. 1). First, we selected the 2,399 genes (type 1 genes) that show light-induced increase of mRNA methylation in the WT plants (WT hyper B/D > 1.5, *P* < 0.05). Second, we identified 1,869 genes (type 2 genes) that showed light-induced increase of translation in the WT and the *mta* mutant but not in the *cry1cry2* and *fio1-1* mutants ((B/D)^WT^ > 1.5, (B/D)^*cry1cry2*^/(B/D)^WT^< 0.8, (B/D)^*fi*^*°*^1^/(B/D)^WT^ < 0.8, (B/D)^*mta*^/(B/D)^WT^ > 0.8, FDR <0.05). Third, we collected 403 genes (type 3 genes) that exhibit higher protein abundance in the light-grown than dark-grown plants of the WT and the *mta* mutant, but lower protein abundance in the light-grown than dark-grown plants of the *cry1cry2* and *fio1-1* mutants ((B/D)^*cry1cry2*^/(B/D)^WT^< 1, (B/D)^*fi*^*°*^1^/(B/D)^WT^ < 1, (B/D)^*mta*^/(B/D)^WT^ > 1, *P* < 0.05). Finally, we used the Venn analysis to identify the overlapping accessions of the three types of genes, resulting in seven such genes (Fig. 2i and Supplementary Table 11). These seven genes showed the statistically significant light-induced increases of m^6^A density (Fig. 2j,k and Extended Data Fig. 2h), translation status (Fig. 2l) and protein abundance (Fig. 2m) in the WT and *mta* mutant, but not in *cry1cry2* and *fio1-1* mutants. We then searched literature for the previous genetics and physiological studies of these seven genes. Remarkably, mutations for six of these seven candidate genes have been previously reported to show a low-chlorophyll phenotype in light-grown plants under various experimental conditions, such that these six genes can indeed be classified as *CHR* genes that regulate chlorophyll homeostasis. Moreover, five of these six *CHR* genes encode chloroplast proteins (Supplementary Table 11)^49–54^, indicating these CHR proteins regulate chlorophyll homeostasis in chloroplasts. These six *CHRs* all showed CRY/FIO1-dependent but MTA-independent light promotion of mRNA methylation, translation and protein abundance in the light-grown plants (Fig. 2 and Supplementary Table 11). This result would explain the genotype-specific chlorophyll phenotypes of the *cry1cry2*, *fio1-1* and *mta* mutants by the mechanism of the writer-specific light activation of mRNA methylation and translation. According to this interpretation, CRYs mediate light activation of FIO1 and MTA, which catalyse light-induced m^6^A deposition to different adenine residues in the same or different mRNA substrates, resulting in differential translation state and protein abundance of the *CHR* genes in response to light, and consequently different chlorophyll contents in the *cry1cry2*, *fio1-1* and *mta* mutants.

### CRY2 interacts with FIO1 in the light-independent manner

**Fig. 3:**
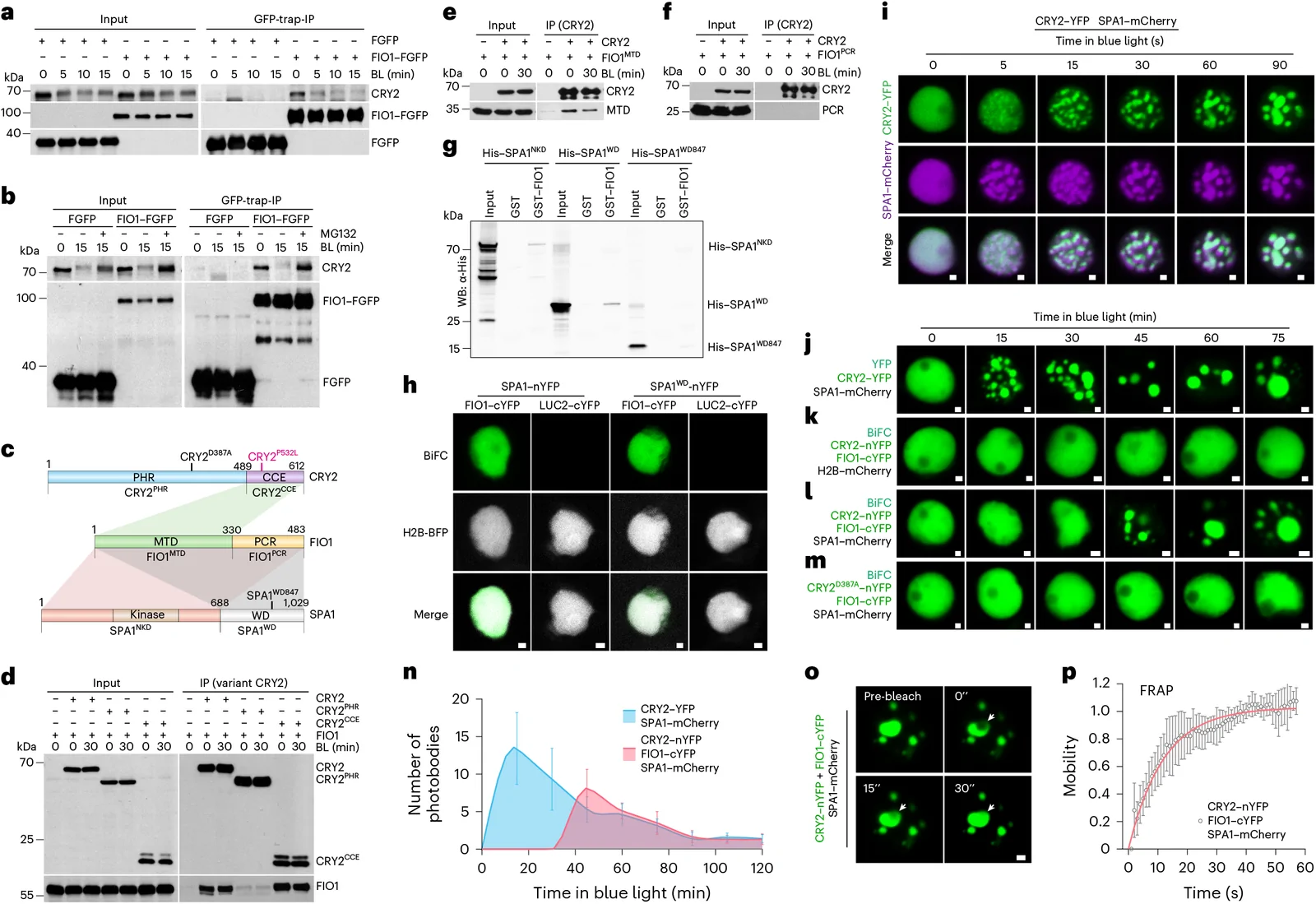
Blue light-induced LLPS of the CRY2/SPA1/FIO1 trimolecular complex. **a**,**b**, Co-IP assays. Six-day-old etiolated seedlings expressing the *pACT2::Flag–FIO1–GFP (FIO1–FGFP)* or *pACT2::Flag–GFP (FGFP)* transgene in WT were exposed to blue light (BL, 25 μmol m^−2^ s^−1^) for indicated time (**a**) or treated with or without MG132 (50 µM) for 4 h before exposure to blue light for 15 min (**b**) before collection. GFP-trap was used for IP. **c**, A diagram showing the interacting domains of CRY2, FIO1 and SPA1. The coloured shades indicate direct protein–protein interaction. **d**–**f**, The co-IP assays show that FIO1 interacts with the CCE domain of CRY2 (**d**), and CRY2 interacts with the MTD domain of FIO1 (**e**), but not the PCR domain of FIO1 (**f**). HEK293T cells co-expressing the indicated proteins were kept in darkness, or exposed to blue light (BL, 100 μmol m^−2^ s^−1^) for 30 min before collection. Flag resin was used for IP. **g**, The in vitro GST pull-down assays showing that SPA1 interacts with FIO1. Glutathione beads bound with GST or GST–FIO1 were incubated with truncated SPA1 proteins. WB, western blot. **h**, BiFC assays showing interaction of SPA1 and FIO1 in tobacco (*N. benthamiana*). LUC2 serves as the negative control, and H2B–BFP serves as the nuclear marker. **i**, Time-lapse co-localization assays showing blue light-induced condensation of CRY2 and SPA1 in CRY2 photobodies. CRY2–YFP and SPA1–mCherry proteins were transiently co-expressed in tobacco leaves. **j**–**m**, Light-induced condensation of CRY2–FIO1 complex in the presence of SPA1. CRY2–YFP co-expressed with SPA1–mCherry (**j**), BiFC pair of CRY2/FIO1 co-expressed with H2B–mCherry (**k**) and CRY2/FIO1 or CRY2^D387A^/FIO1 BiFC pairs co-expressed with SPA1–mCherry (**l** and **m**) in tobacco. The signals in the nucleus were detected for the indicated time. **n**, Quantification of the photobody number over the time for blue-light illumination in the assays shown in **j** and **l**. The data are shown as mean ± s.d. **o**, FRAP analysis of CRY2–FIO1 condensates in the presence of SPA1 in tobacco leaf cells. The representative images showing the region (white arrowhead) before and after photobleaching. **h**–**o**, Scale bar, 2 μm. **p**, Quantification of the FRAP assay shown in **o**. The double exponential fit (red line) of averaged recovery curves is shown (mean ± s.d.; *n* = 5 independent experiments). Source data

**Extended Data Fig. 4:**
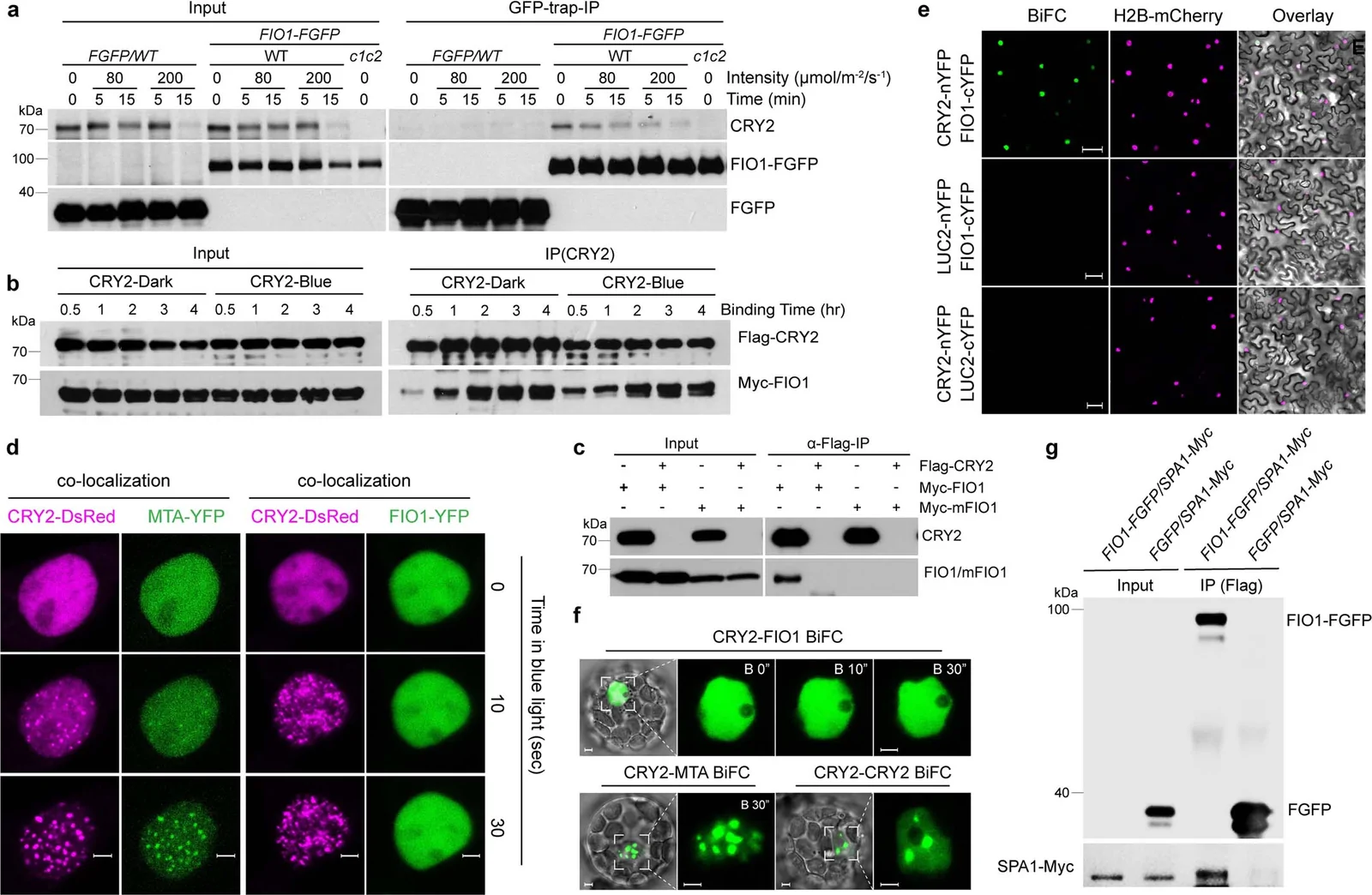
CRY2 interacts with SPA1 and FIO1 to recruit FIO1 into the CRY2 photobodies. **a**. The co-IP assay showing the interaction of FIO1 and CRY2 in plants. The 6-day-old etiolated *pACT2::Flag-FIO1-GFP (FIO1-FGFP) or pACT2::Flag-GFP (FGFP)* transgenic plants exposed to white light with different intensities (0, 80, and 200 μmol m^−2^ s^−1^) for the indicated time. GFP-trap was used for IP. **b**. The co-IP assay showing the interaction of Myc-FIO1 and Flag-CRY2 in heterologous HEK293T cells. Cells co-expressing the indicated proteins were kept in the dark and lysed, anti-Flag affinity beads were then incubated with the cell lysates in the dark or blue light (BL, 100 μmol m^−2^ s^−1^) for indicated time. Anti-Flag beads were used for IP. **c**. The co-IP assay showing the disrupted interaction of Flag-tagged CRY2 and Myc-tagged FIO1 mutant (Myc-FIO1 or Myc-mFIO1) in heterologous HEK293T cells. Cells co-expressing the indicated proteins were kept in the dark. Flag resin was used for IP. **d**. The co-localization analysis of CRY2-DsRED and MTA-YFP or FIO1-YFP in heterologous HEK293T cells. Cells co-expressing the indicated proteins were kept in the darkness or exposed to blue light at the indicated time. Scale bar= 2 μm. **e**. The BiFC assays showing FIO1-CRY2, MTA-CRY2 and CRY2-CRY2 interactions in response to blue light (2% of the 488 nm laser power) for indicated time in Arabidopsis protoplasts. Scale bar= 2 μm. **f**. The BiFC assays show FIO1-CRY2 interaction in tobacco leaves under blue light. Scale bar= 50 μm.H2B-mCherry served as a nuclear marker. LUC2 was used as the negative control for BiFC assays. **g**. The co-IP assay showing the interaction of FIO1 and SPA1 in plants. The 6-day-old *pACT2::Flag-FIO1-GFP* (*FIO1-FGFP*) x *35S::SPA1-Myc* (SPA1-Myc) or *pACT2::Flag-GFP* (*FGFP*) x *35S::SPA1-Myc* transgenic plants were grown under blue light (25 μmol m^−2^ s^−1^). Flag resin was used for IP. Immunoblotting was with anti-Flag or anti-Myc antibody. Source data

To further investigate the mechanisms underlying blue-light regulation of the FIO1-dependent mRNA methylation, we examined whether CRY2 may complex with FIO1 in vivo. In this experiment, *Arabidopsis* seedlings grown in the dark were transferred to blue light (25 μmol m^−2^ s^−1^), and the possible CRY2/FIO1 complex was examined by co-immunoprecipitation (co-IP) assay. Figure 3a shows that CRY2 complexes with FIO1 in *Arabidopsis* seedlings in the light-independent manner. The seemingly reduced amount of the endogenous CRY2 pulled down by the recombinant FIO1 from blue light-grown seedlings was due to the blue light-induced and 26S proteome-dependent CRY2 degradation (Fig. 3b and Extended Data Fig. 4a)^55,56^. We next used co-IP assay to examine whether CRY2 may directly interact with FIO1 in the mammalian HEK293 cells co-expressing the CRY2 and FIO1 recombinant proteins (Fig. 3c–f and Extended Data Fig. 4b). The CRY2/FIO1 complex was detected in HEK293 cells regardless of blue light treatment. Because HEK293 cells lack plant proteins that might cause indirect interaction of CRY2 and FIO1, we concluded that, similar to MTA, CRY2 physically interacts with FIO1 in a light-independent manner. CRY2 has two functional domains (Fig. 3c), the N-terminal photolyase homologous region (PHR) domain that binds to the chromophore flavin adenine dinucleotide (FAD) for photon absorption, and the CRY C-terminal Extension (CCE) domain that is an intrinsically disordered region (IDR) required for CRY2 or the CRY2/MTA complex in the liquid phase when they undergo light-induced LLPS^15^. FIO1 also has two domains (Fig. 3c), the N-terminal methyl transferase domain (MTD) that is conserved in all METTL16-like writers and the C-terminal plant conserved region (PCR) domain that is highly conserved in the FIO1 paralogues from green algae to flowering plants^40^. Results of co-IP assays, using HEK293 cells co-expressing various versions of the CRY2 and FIO1 recombinant proteins, demonstrate that the CCE domain of CRY2 physically interacts with the MTD domain of FIO1 (Fig. 3d–f and Extended Data Fig. 4c). For example, the CCE domain (CRY2^CCE^) but not the PHR domain of CRY2 (CRY2^PHR^) pulled down FIO1 (Fig. 3d), whereas the MTD domain of FIO1 but not the PCR domain (FIO1^PCR^) of FIO1 pulled down CRY2 (Fig. 3e,f). The FIO1 mutant (mFIO1) protein, which resembles the *fio1-1* mutant allele by in-frame deletion of five amino acids (D^145^FTVV^149^) in the MTD domain of FIO1 (ref. ^36^), failed to interact with CRY2 (Extended Data Fig. 4c). These results demonstrate the domain specificity of the interaction between CRY2 and FIO1. We also examined interaction of CRY2 to FIO1 or MTA, using the co-localization assay in HEK293 cells expressing the fluorescence-labelled recombinant CRY2 and FIO1 or CRY2 and MTA (Extended Data Fig. 4d) or bimolecular fluorescence complementation (BiFC) assay in *Arabidopsis* protoplasts or tobacco (*Nicotiana benthamiana*) leaves transiently expressing the respective proteins (Extended Data Fig. 4e,f). To our surprise, the CRY2/MTA complex, but not the CRY2/FIO1 complex, showed light-induced condensation. For example, the CRY2–DsRED and FIO1–YFP recombinant proteins showed no photoresponsive condensation like that of the CRY2/MTA complex (Extended Data Fig. 4d). Similarly, the BiFC signals of the CRY2–nYFP/FIO1–cYFP complex was detected in the nucleoplasm of *Arabidopsis* protoplasts or tobacco (*N. benthamiana*) leaf cells transiently expressing CRY2–nYFP/FIO1–cYFP, confirming their physical interaction, but no obvious photoresponsive condensation of the BiFC signals was observed (Extended Data Fig. 4e,f). In contrast, photoexcited CRY2 co-condensed MTA into the CRY2 photobody within 30 s upon blue-light illumination (Extended Data Fig. 4f)^15^. We speculated that the CRY2/FIO1 complex might respond to blue light only in the presence of a photoresponsive CRY2-interacting protein that is absent in HEK293 cells or present at a too low concentration in the plant cells tested to allow observation of photoresponsive condensation of the CRY2/FIO1 complex.

### The blue light-dependent LLPS of the CRY2/FIO1/SPA1 complex

We speculated that the CRY2-signalling protein SPA1 may act as a chaperone to facilitate photoresponsive actions of the CRY2/FIO1 complex, because SPA1 is a major CRY2-signalling protein and it is a light-dependent chaperone that recruits photoexcited CRYs to the E3 ligase CUL4^COP1/SPA1^ to inhibit the activity of COP1 (refs. ^12,14,57,58^). To test this possibility, we first analysed whether SPA1 may physically interact with FIO1. Due to technical difficulties in purifying the full-length SPA1 protein, we expressed and purified individual SPA1 domains or mutant controls in *Escherichia coli*, purified the recombinant proteins and examined their interaction with FIO1, using the in vitro pull-down assay. Results of this experiment show that both the N-terminal kinase domain (SPA1^NKD^) and the C-terminal WD domain of SPA1 (SPA1^WD^) physically interact with FIO1 in vitro with the modest affinity (Fig. 3g). To investigate the specificity of the SPA1–FIO1 interaction, we examined the mutant WD domain of SPA1 (SPA1^WD847^) that resembles the loss-of-function *spa1-1* mutant allele by deletion of the last 182 residues of the C-terminus of SPA1 (ref. ^10^). Figure 3g shows that the mutant WD domain of SPA1 had markedly lower affinity to FIO1, supporting the notion that SPA1 specifically interacts with FIO1. We also confirmed the SPA1–FIO1 interaction in plant cells, using the BiFC assay (Fig. 3h) and the co-IP assay (Extended Data Fig. 4g). The clear BiFC signal resulting from reconstitution of the FIO1–nYFP and SPA^WD^–cYFP recombinant proteins confirms that the WD domain of SPA1 physically interacts with FIO1 to form the SPA1/FIO1 complex (Fig. 3h). Together, these results argue for possible existence of a photoresponsive CRY2/SPA1/FIO1 trimolecular complex, in which FIO1 may be activated in response to light without altering its affinity to CRY2 or SPA1.

**Fig. 4:**
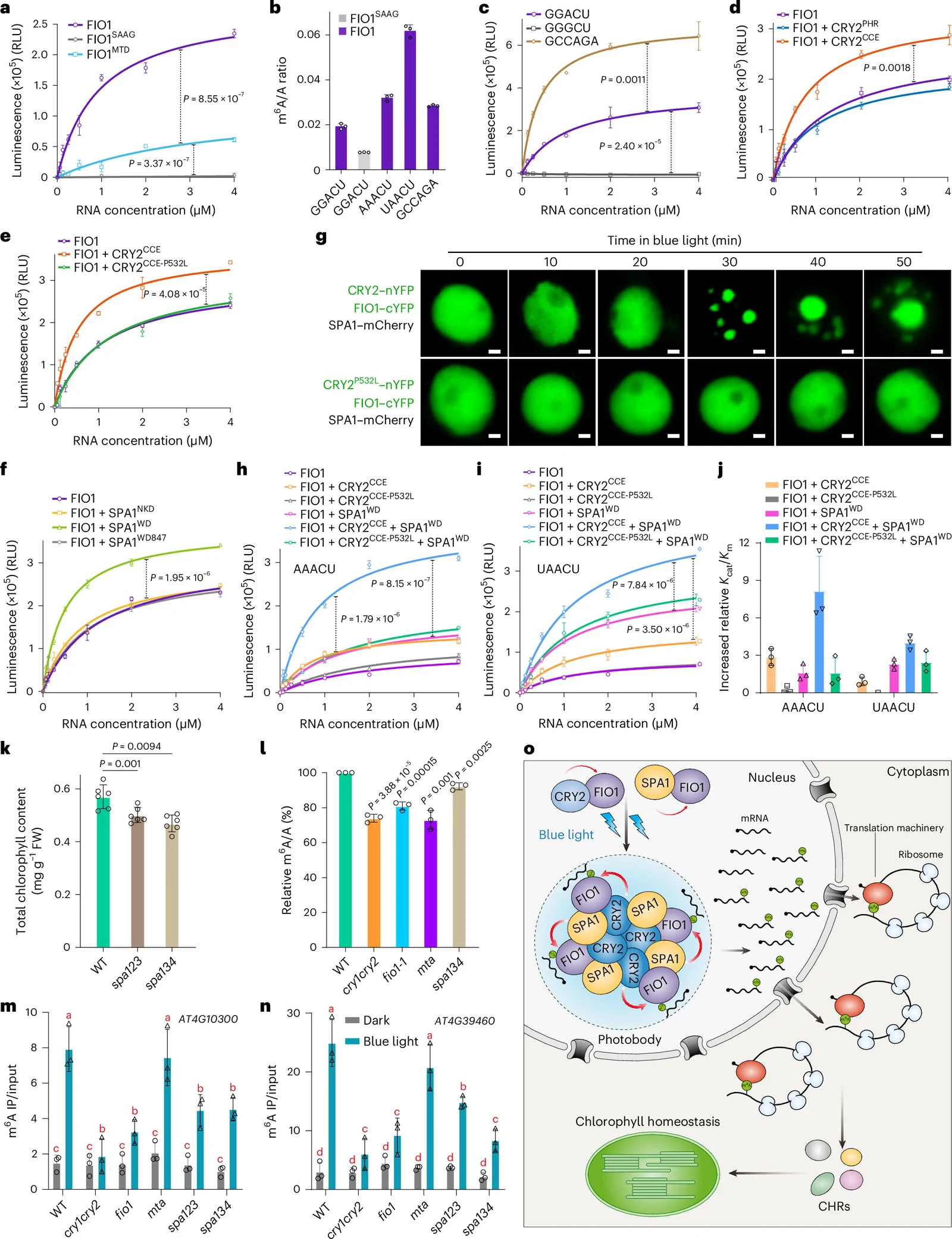
CRY2 and SPA1 synergistically activate FIO1 in vitro. **a**, Steady-state kinetics of m^6^A methylation by FIO1, FIO1^SAAG^ and FIO1^MTD^. RLU, relative light unit. **b**, LC–MS/MS analysis of the relative level of m^6^A in total adenosine (m^6^A/A). Different RNAs incubated with either FIO1 or FIO1^SAAG^ from the in vitro m^6^A methylation assay were purified for m^6^A levels by LC–MS/MS. **c**, Steady-state kinetics of m^6^A installation by FIO1 on GGACU, GGGCU and GCCAGA substrates. **d**–**f**, Steady-state kinetics of m^6^A methylation catalysed by FIO1 with or without 2 μM proteins indicated for CRY2^PHR^ or CRY2^CCE^ (**d**), CRY2^PHR^ or CRY2^CCE^ (**e**) and various versions of SPA1 proteins (**f**). **g**, BiFC assays showing the CRY2/FIO1 or CRY2^P532L^/FIO1 complex in the presence of SPA1–mCherry in response to blue light at the indicated time in tobacco. Scale bar, 2 μm. **h**,**i**, Steady-state kinetics of m^6^A methylation of AAACU (**h**) or UAACU (**i**) by FIO1 with or without 2 μM indicated effector proteins. **j**, Effects of the CRY2 and SPA1 protein fragments on the catalytic efficiency (*K*_cat_/*K*_m_) of FIO1. The increased *K*_cat_/*K*_m_ represents the difference between the *K*_cat_/*K*_m_ of FIO1 with effector proteins and its basal value (set as 1.0). The data **a**–**j** are presented as mean ± s.d. (*n* = 3 independent experiments), and *P* values are from two-tailed Student’s *t*-test. **k**, Total chlorophyll contents of 6-day-old seedlings grown under blue light (25 μmol m^−2^ s^−1^). Mean ± s.d. (*n* = 5 independent experiments). **l**, LC–MS/MS analysis of the relative level of m^6^A (m^6^A/A) in mRNA purified from seedlings in blue light (25 μmol m^−2^ s^−1^) for 6 days. m^6^A/A ratio for each genotype was normalized to that of WT (mean ± s.d., *n* = 3 independent experiments). *P* values are from two-tailed Student’s *t*-test. **m**,**n**, The m^6^A level of m^6^A peaks detected by MeRIP-seq was analysed by m^6^A-IP–quantitative polymerase chain reaction. Six-day-old seedlings grown in blue light (25 μmol m^−2^ s^−1^) or darkness were used in the assay. The lowercase letters indicate statistically significant differences (*P* < 0.05) by one-way ANOVA test followed by Tukey’s multiple comparisons test (mean ± s.d., *n* = 3 independent experiments). The exact *P* values are provided in Supplementary Table 16. **o**, A hypothetical model depicting the regulatory mechanism of chlorophyll homeostasis by CRY2/FIO1/SPA complex. Source data

**Extended Data Fig. 5:**
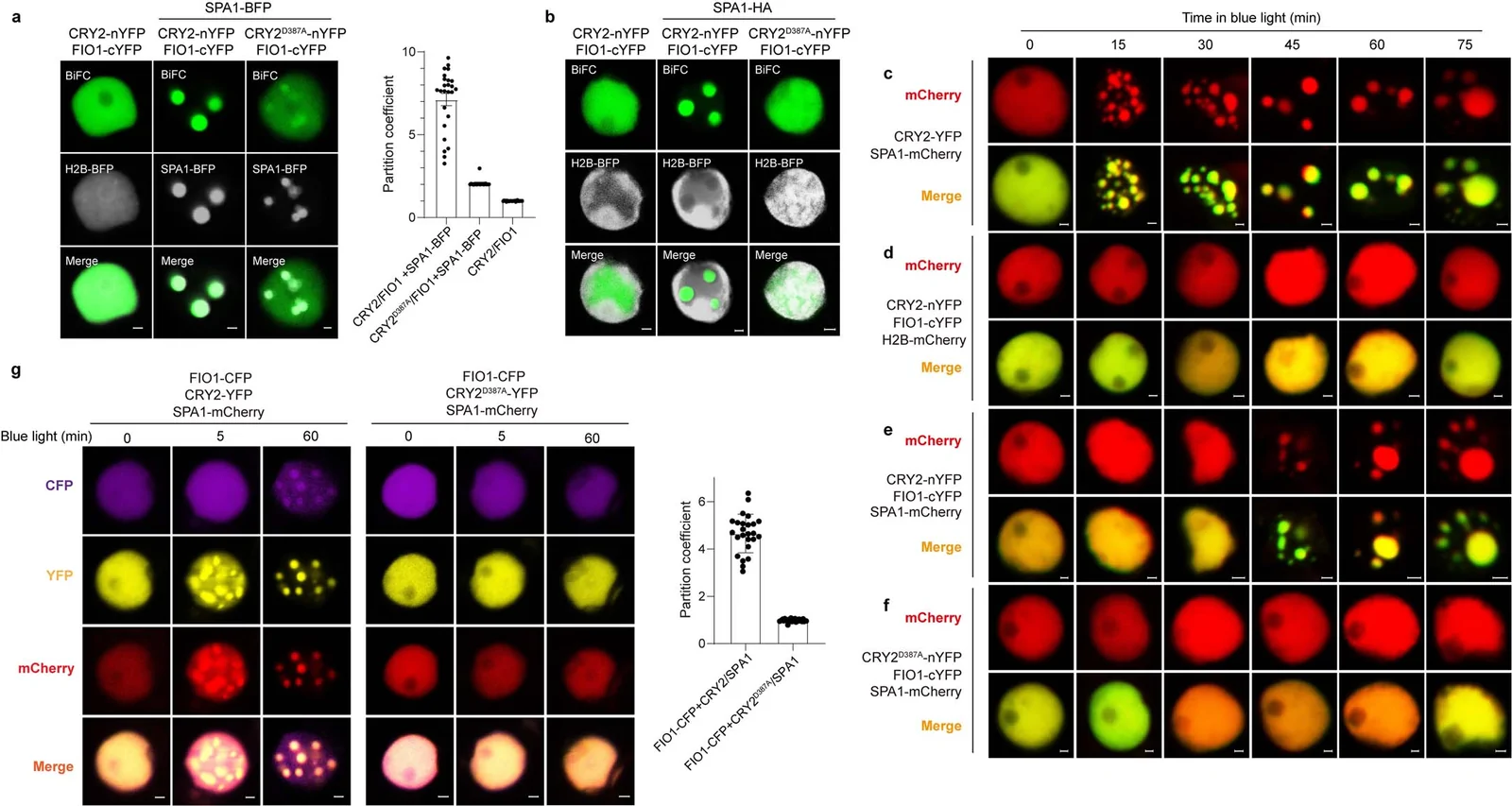
FIO1 co-localized with SPA1 and CRY2 in photobodies. **a**. Left, the BiFC assays show the formation of CRY2-FIO1 or CRY2^D387A^-FIO1 condensates in the presence of SPA1-BFP in tobacco leaf cells under blue light (2% of the 488 nm laser power). Scale bar, 2 μm. Right, the partition co-efficient of CRY2-FIO1 or CRY2^D387A^-FIO1 co-expression with SPA1-BFP in tobacco leaf cells. (mean ± s.d., n = 25 measurements from five nuclei). **b**. The BiFC assays show the formation of CRY2-FIO1 condensates in the presence of SPA1-HA in tobacco leaf cells under blue light (2% of the 488 nm laser power for 1 h). Light-insensitive CRY2^D387A^-FIO1 complex was used as the control. Scale bar, 2 μm. **c-f**. Light-induced condensation of CRY2-FIO1 complex in the presence of SPA1. CRY2-YFP co-expressed with SPA1-mCherry (**c**), BiFC pair of CRY2/FIO1 co-expressed with H2B-mCherry (**d**) and CRY2/FIO1 or CRY2^D387A^/FIO1 BiFC pairs co-expressed with SPA1-mCherry (**e-f**) in tobacco leaf cells. The CRY2-YFP or CRY2-FIO1 BiFC signals in the nucleus were detected by confocal microscopy with 488 nm laser (2% of laser power) as the light source to induce the formation of CRY2 or CRY2-FIO1 condensates for the indicated time. Scale bar=2 μm. **g**. Left, images showing the partial colocalization of FIO1, CRY2 and SPA1 in CRY2 photobodies over the time of blue laser illumination in tobacco leaf cells. CRY2^D387A^-FIO1-SPA1 complex was used as the control. Scale bar, 2 μm. Right, the partition co-efficient of FIO1-CFP co-expression with CRY2/SPA1 or CRY2^D387A^/SPA1 in tobacco leaf cells. (mean ± s.d., n = 25 measurements from five nuclei). Source data

To directly test how SPA1 affects light responses of the CRY2/FIO1 complex, we compared effects of blue light on the CRY2/CRY2, CRY2/SPA1 and CRY2/FIO1 complexes in tobacco leaves transiently expressing or co-expressing the recombinant proteins of CRY2 (CRY2–YFP and CRY2–nYFP), FIO1 (FIO1–cYFP and FIO1–CFP) and SPA1 (SPA1–mCherry, SPA1–HA or SPA1–BFP), using the BiFC and fluorescence co-localization assays. CRY2–nYFP and FIO1–cYFP recombinant proteins clearly interact to reconstitute YFP emitting the BiFC signal, but no condensation was detected for the CRY2/FIO1 complex in the absence of SPA1 (Fig. 3k and Extended Data Fig. 5a,b,d). As expected, blue light induces rapid (~5 s) condensation of the CRY2/SPA1 complex to the CRY2 photobody (Fig. 3i,j and Extended Data Fig. 5c). Importantly, blue light also induced condensation of the CRY2/FIO1 complex to CRY2 photobody in plant cells co-expressing any of the three SPA1 recombinant proteins tested, including SPA1–mCherry (Figs. 3l,o and 4g), SPA1–BFP (Extended Data Fig. 5a) and SPA1–HA (Extended Data Fig. 5b). These results demonstrate that SPA1 acts as a chaperone facilitating the photoresponsive co-condensation of the FIO1/CRY2 complex. Results of the co-localization assays also demonstrate photoresponsive co-condensation of the CRY2/SPA1/FIO1 trimolecular complex (Extended Data Fig. 5e,g). Kinetically, the light-dependent condensation of the CRY2/SPA1/FIO1 trimolecular complex occurred slowly, which did not appear until ~30 min after blue-light illumination (Fig. 3n). This is in stark contrast to the rapid (within 5 s) light induction of condensation of the CRY2/CRY2 (Fig. 3i,j), CRY2/MTA (Extended Data Fig. 4d,f) or CRY2/SPA1 (Fig. 3i) complexes. Among the three SPA1 fusion proteins tested, SPA1–mCherry and SPA1–HA do not form nuclear body in the absence of CRY2, and they promote co-condensation of FIO1 with CRY2 but not co-condensation of FIO1 with the photo-inactive CRY2^D387A^ mutant (Extended Data Fig. 5b,d,g). SPA1–BFP promotes co-condensation of FIO1 to not only photoactive CRY2 but also the photo-inactive CRY2^D387A^ mutant, albeit at lower efficiency (Extended Data Fig. 5a). Because the CRY2^D387A^ mutant does not interact with SPA1 (ref. ^56^) and the SPA1–BFP-dependent CRY2^D387A^/FIO1 condensates exhibit much lower partition coefficient than that of the SPA1–BFP-dependent CRY2/FIO1 condensates (Extended Data Fig. 5a, right), these results are consistent with the notion that the SPA1–CRY2 and SPA1–FIO1 interactions are both required for the photoresponsive co-condensation of the CRY2/FIO1/SPA1 trimolecular complex, and that SPA1 acts as the light-dependent chaperone that facilitates the gradual co-condensation of the CRY2/SPA1/FIO1 trimolecular complex in response to blue light.

**Extended Data Fig. 6:**
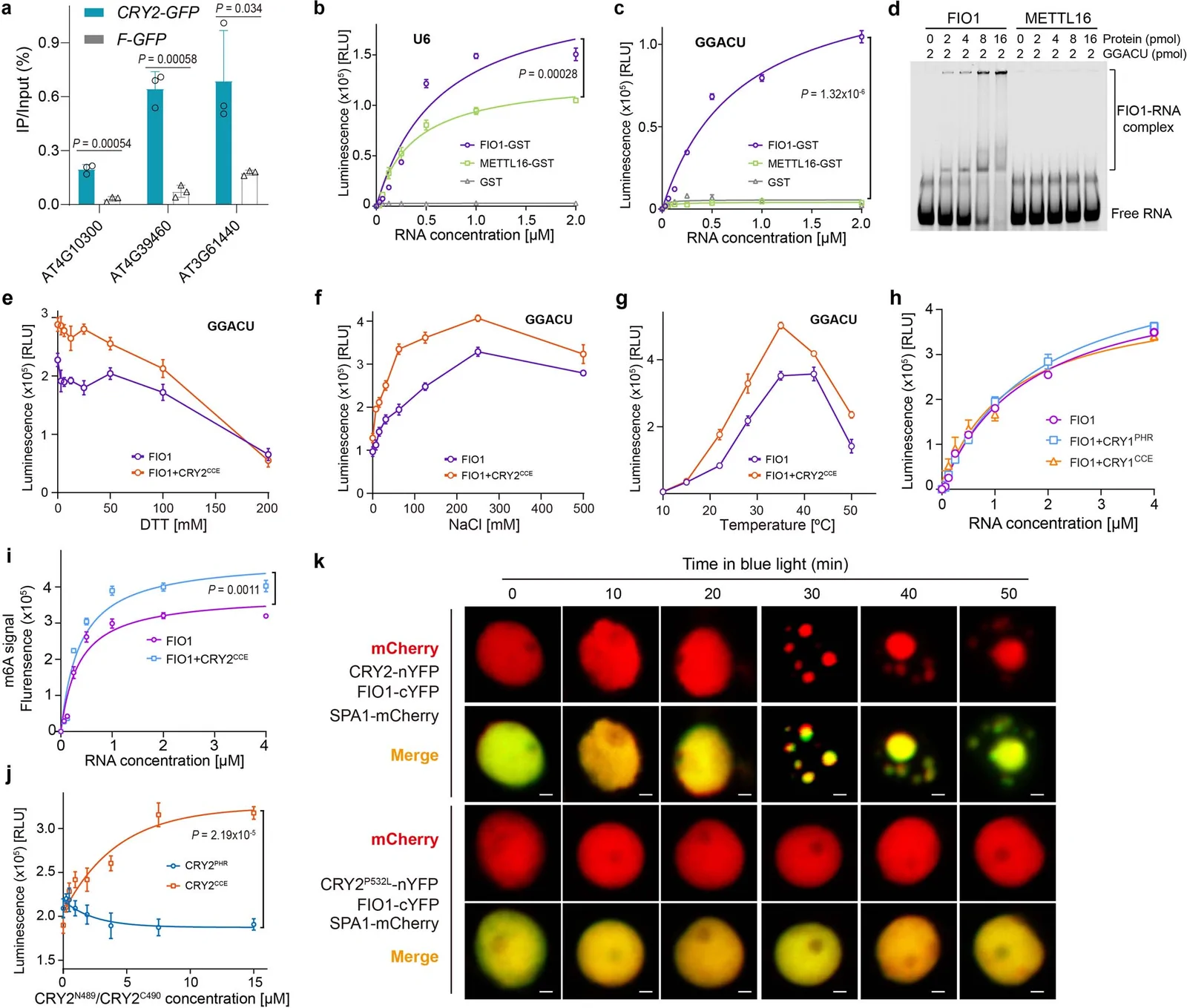
CRY2 enhances FIO1 enzymatic activity *in vitro* via the SPA1-interacting CCE domain. **a**. The native RIP assay allows for direct identification of bound RNAs to CRY2 under blue light (25 μmol m^−2^ s^−1^). The 6-day-old *CRY2-GFP* and *F-GFP* transgenic plants were grown under blue light. GFP trap resin were used for IP. 10% of the extract was used as input. **b-c**. Steady-state kinetics of m^6^A methylation of U6 snRNA (**b**) and GGACU RNA (**c**) by FIO1(1 μM), METTL16 (1 μM), and GST (1 μM). **d**. RNA-binding assay showing that FIO1, but not MELL16 binds to GGACU RNA. **e-g**. Characterization of enzymatic activities of FIO1 (1 μM) or FIO1(1 μM) + CRY2^CCE^ (2 μM) for installing m^6^A in GGACU (2 μM) under various conditions, including different DTT concentrations (**e**), different NaCl concentrations (**f**) and different temperatures (**g**). **h**. FIO1-catalyzed m^6^A methylation of different concentrations of GGACU in the presence or absence of CRY1^PHR^ (2 μM) or CRY1^CCE^ (2 μM). **i**. m^6^A antibody-based ELISA assays showing the steady-state kinetics of m^6^A installation in GGACU by FIO1(1 μM), and FIO1(1 μM) + CRY2^CCE^ (2 μM). **j**. The dosage-dependent influences of CRY2^PHR^ and CRY2^CCE^ on the enzymatic activities of FIO1(1 μM) for installing m^6^A in GGACU RNA. Data (**a-j**) is presented as mean ± s.d. (n = 3 independent experiments). P values are determined from two-tailed Student’s *t*-test. **k**. BiFC assays showing the changes of nuclear CRY2/FIO1 or CRY2^P532L^/FIO1 complex in the presence of SPA1-mCherry in response to blue light at the indicated time in tobacco leaf cells. Scale bar=2 μm. Source data

To further examine the CRY2/SPA1/FIO1 trimolecular complex, we analysed how the light-insensitive and physiologically inactive CRY2^D387A^ mutant^59^ interacts with FIO1 in plant cells, using the BiFC assays. We observed clear BiFC fluorescence signals in plant cells co-expressing CRY2^D387A^–nYFP, FIO1–cYFP and SPA–HA or SPA1–mCherry, regardless of light treatment (Fig. 3m and Extended Data Fig. 5b,f). This result demonstrates that the CRY2^D387A^ mutant can physically interact with FIO1 in the light-independent manner, which is consistent with the light-independent nature of the CRY2–FIO1 interaction. But only the wild-type CRY2 fusion proteins co-condensed FIO1 in the presence of SPA1, confirming that only the photochemically active CRY2 can facilitate blue light-induced condensation of the CRY2/SPA1/FIO1 complex. Because the CRY2^D387A^ is physiologically inactive, this result also demonstrates the physiological relevance of the photoresponsive CRY2/SPA1/FIO1 condensation. We next tested whether the CRY2/SPA1/FIO1 condensates are in the biochemically active liquid phase or biochemically inactive non-liquid aggregates (Fig. 3o–p), using the fluorescence recovery after photobleaching (FRAP) assay as we described previously^15^. In this experiment, we co-expressed CRY2–nYFP, FIO1–cYFP and SPA1–mCherry in tobacco leaves, exposed leaves to green light (514 nm laser) that excites YFP, selected cells showing the BiFC fluorescence signal of the CRY2–nYFP/FIO1–cYFP complex, illuminated the cells with blue light (488 nm laser) and quantified recovery of the BiFC signals of the CRY2/SPA1/FIO1 condensates after photobleaching. Results of this FRAP experiment show that more than 80% of the BiFC signal of the CRY2/SPA1/FIO1 condensates rapidly recovered within 20 s after laser bleach (Fig. 3p), demonstrating that the CRY2/SPA1/FIO1 condensates are in the physiologically active liquid phase. We also investigated whether mRNA may be recruited to the CRY2/SPA1/FIO1 condensates, using the RNA immunoprecipitation (RIP)–quantitative polymerase chain reaction assays. The results of this experiment show that the CRY2–GFP recombinant protein in the transgenic plants is physically associated with at least the three *CHR* transcripts tested, *AT4G10300*, *AT4G39460* and *AT3G61440* (Extended Data Fig. 6a). These results are consistent with the hypothesis that blue light induces condensation of the CRY2/SPA1/FIO1 trimolecular complex to directly activate FIO1 and mRNA methylation.

### CRY2 and SPA1 cooperatively activate FIO1 in vitro

To further test the hypothesis that the CRY2/SPA1/FIO1 trimolecular complex may activate FIO1, we characterized the m^6^A RNA methyltransferase activity of FIO1 expressed and purified from *E. coli*, using the MTase-Glo Methyltransferase Assay (Promega) and liquid chromatography–tandem mass spectrometry (LC–MS/MS) (Fig. 4). Figure 4a shows that the MTD domain of FIO1 alone is catalytically active, although its activity is markedly lower than that of the full-length FIO1 protein. The mutation of FIO1^SAAG^, of which the four residues (NPPF) at the catalytic site of FIO1 are replaced by SAAG, almost completely abolishes the enzymatic activity of FIO1 (Fig. 4a,b). FIO1 is capable of catalysing m^6^A deposition to various RNA substrates that bear the RRACH (GGACU, AAACU or UAACU) or the related YHAGA (GCCAGA) sequences, although different substrates exhibit different efficiency of m^6^A deposition catalysed by FIO1 in vitro (Fig. 4b,c). The RNA substrates lacking the A residue or deviating from the canonical RRACH motif are unmethylated (Fig. 4c). FIO1 structurally resembles the metazoan METTL16, but FIO1 binds to both U6 and the RRACH-bearing RNA substrates and deposits m^6^A to both types of RNA substrate (Extended Data Fig. 6b–d). In contrast, human METTL16 binds to and methylates U6 RNA but it does not bind or methylate the other RNA substrates tested (Extended Data Fig. 6b–d). Our results that FIO1 can deposit the m^6^A mark on a wide range of RNA substrates (Fig. 4b and Extended Data Figs. 3g–i and 6b–d) are consistent with the previous studies^40–43^. Results of the in vitro enzymatic activity assays also show that FIO1 methyltransferase activity varied in response to different oxidative, salt and temperature conditions (Extended Data Fig. 6e–g), providing possible explanation of how plant cells alter mRNA methylation in responses to different environmental conditions^18^.

**Extended Data Fig. 7:**
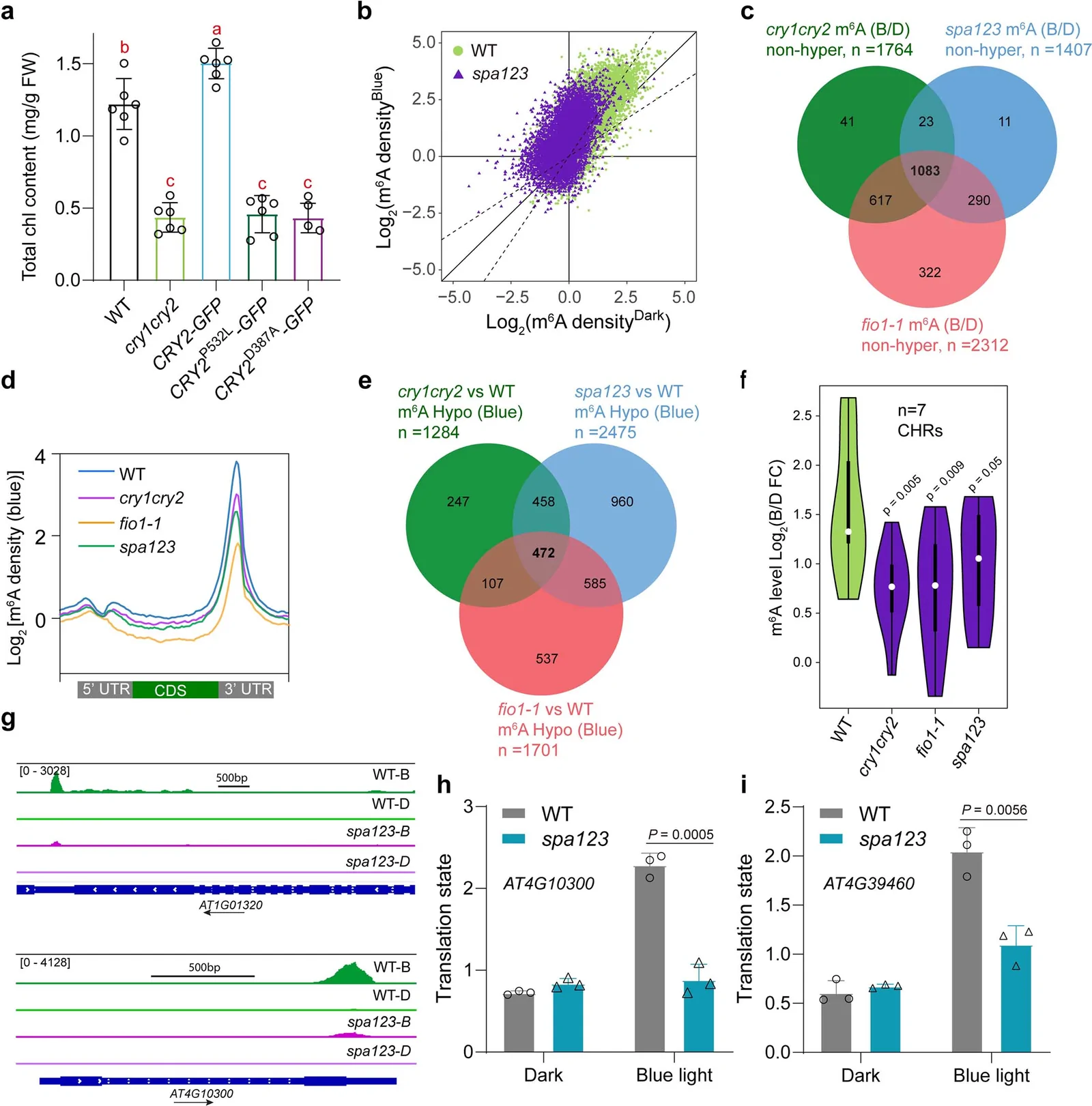
SPA1 is required for blue light induced m^6^A methylation. **a**. Total chlorophyll content of 6-day-old plants grown under blue light (25 μmol m^−2^ s^−1^) (mean ± s.d., n = 6 independent experiments). The lowercase captions of different letters indicate statistically significant differences (*P* < 0.05) by one way ANOVA test followed by Tukey’s multiple comparisons test. **b**. Scatter plots showing the photoresponsive epitranscriptomic changes of the same 8-sample cohort as in Fig. 2b. The ‘m^6^A density’ represents the number of m^6^A detected per unit length of RNA sequenced in the wild-type (WT, green) or *spa123* mutant (purple) grown in dark or blue light (25 μmol m^−2^ s^−1^) for 6 days before harvest. The dashed lines indicate >1.5x fold change. **c**. The Venn graph showing overlaps of photoresponsive m^6^A epitranscriptome of the indicated genotypes (FC^B/D^ > 1.5, *P* value < 0.05, two-tailed Student’s *t*-test). The ‘m^6^A non-hyper’ are defined by m^6^A-B/D^WT^ > 1.5 & m^6^A-B/D^mt^ < 1.5, WT: wild-type, mt: mutants. **d**. The m^6^A intensity of the indicated genotypes grown in blue light are mapped to the transcriptome. **e**. The Venn graph showing overlaps of hypomethylated (m^6^A hypo, FC^B/D^ > 1.5, *P* value < 0.05, two-tailed Student’s *t*-test) peaks indicated genotypes grown in blue light. **f**. Violin plots comparing the m^6^A density of *CHR* genes (Fold change of blue vs dark, B/D FC, n = 7 genes) in WT and indicated mutants. The upper, middle, and lower segments represent the dataset’s upper quartile, median, and lower quartile, respectively. **g**. The m^6^A density maps of the representative CHR transcripts. **h-i**. Results of Ribo-tag IP-quantitative polymerase chain reaction assays showing the translation states of WT and *spa123* mutant. 6-day-old seedlings grown in darkness or blue light (25 μmol m^−2^ s^−1^) were harvested for analysis. Ribosomes were immunoprecipitated by anti-FLAG resin and the associated RNA were quantified by quantitative polymerase chain reaction (mean ± s.d., n = 3 independent experiments). *P* values are determined from two-tailed Student’s *t*-test. Source data

We next investigated how CRY2 and SPA1 affect the enzymatic activity of FIO1 and obtained the following results (Fig. 4d–i). First, CRY2 possesses the FIO1-activating activity. The FIO1-interacting CCE domain of CRY2 (CRY2^CCE^), but not the photon-absorbing PHR domain of CRY2 (CRY2^PHR^), can stimulate the m^6^A RNA methyltransferase activity of FIO1 in vitro (Fig. 4d and Extended Data Fig. 6i,j). It is not clear whether CRY1 may directly affect FIO1 activity. Although we did not detect a stimulatory activity of CRY1^PHR^ and CRY1^CCE^ on the FIO1 activity in vitro (Extended Data Fig. 6h), inappropriate selection of the truncated CRY1 sequences or poor folding of the purified proteins cannot be excluded. Second, the WD domain of SPA1 possesses the FIO1-activating activity in vitro (Fig. 4f). Although both the WD domain and NKD domain of SPA1 interact with FIO1 (Fig. 3g), only the WD domain of SPA1 (SPA1^WD^) (Fig. 3i,j), but not the NKD domain of SPA1 (SPA1^NKD^), activates the m^6^A writer activity of FIO1 in vitro (Fig. 4f). Third, the bi-residue VP motif of CRY2 is essential for both the FIO1-activating activity and FIO1-condensing activity of CRY2 (Fig. 4e–g). The VP motif is composed of valine^531^ and proline^532^, and it is in the CCE domain of CRY2 (Fig. 3c)^14,60^. The VP motif is essential for the light signal transduction but not the light signal perception, nor the overall structural integrity of CRY2. CRY2^P532L^ mutant loses its physiological activities but still retains its photochemical activities, such as photoresponsive oligomerization and photo-condensation activities^14,56,60^. Figure 4e shows that, in contrast to the WT CCE domain of CRY2, the CCE domain of CRY2 impaired in the VP motif (CRY2^CCE-P532L^) failed to activate FIO1. Importantly, the VP motif of CRY2 is also essential for the light-induced CRY2/SPA1/FIO1 co-condensation in plant cells. Figure 4g shows that the CRY2^P532L^ mutant still physically interacts with FIO1, because the BiFC fluorescence signals were detected in plant cells co-expressing the CRY2^P532L^–nYFP and FIO1–cYFP recombinant proteins (Fig. 4g and Extended Data Fig. 6k). However, in contrast to the WT CRY2 protein, the CRY2^P532L^ mutant failed to co-condense FIO1 in plant cells in the presence of SPA1 (Fig. 4g). Consistently, transgenic expression of the CRY2^D387A^ and CRY2^P532L^ mutant proteins failed to rescue the low-chlorophyll phenotype of *cry1cry2*, confirming that the VP motif-dependent CRY2 condensation is required for the CRY2 function in vivo (Extended Data Fig. 7a). Fourth, CRY2 and SPA1 synergistically or additively activate FIO1. Because the CCE domain of CRY2 interacts with the WD domain of SPA1 (ref. ^14^), we tested how the CCE domain of CRY2 and the WD domain of SPA1 activate FIO1 (Fig. 4h–j). We selected two different RNA sequences that bear different RRACH-like motifs, AAACU and UAACU. AAACU is found near the stop codon and 3′ UTR of four *CHRs*; UAACU is found near the stop codon and 3′ UTR of three *CHR*s we identified (Fig. 2j). The CCE domain of CRY2 and the WD domain of SPA1 can act alone to activate FIO1 in vitro (Fig. 4h–j). We further analysed how the individual effector affected the catalytic efficiency (*K*_cat_/*K*_m_) of FIO1. Figure 4j shows that, by taking into account the effector concentration, the CCE domain of CRY2 and the WD domain of SPA1 alone increased the catalytic efficiency of FIO1 by a factor of about 1–3 for the two RNA substrates tested, but including the CCE domain of CRY2 and the WD domain of SPA1 in the same reaction increased the catalytic efficiency of FIO1 by a factor of about 4–8 for the two RNA substrates tested. It is conceivable that depending on the structure of different mRNAs, the CRY2/SPA1/FIO1 complex may activate FIO1 additively or synergistically in vivo. Importantly, the CRY2^CCE-P532L^ mutant, which has diminished SPA1- or FIO1-interacting activity, failed to activate FIO1 in vitro (Fig. 4h–j). Results of these experiments indicate that the light-induced co-condensation of the CRY2/SPA1/FIO1 trimolecular complex activates the enzymatic activity of FIO1 to promote mRNA methylation in response to light.

### SPA1 positively regulates m^6^A deposition

According to our hypothesis, SPA1 would act together with CRY2 to positively regulate light-dependent mRNA methylation and to maintain chlorophyll homeostasis. To test this, we first analysed chlorophyll content of the two previously reported *spa* triple mutants, *spa123* and *spa134* (ref. ^61^). Figure 4k shows that both *spa123* and *spa134* mutants grown in blue light exhibited a low-chlorophyll phenotype in comparison with the WT control. We next examined how the *spa123* and/or *spa134* triple mutants affect mRNA methylation. The LC–MS/MS analyses show that, similar to the *cry1cry2*, *fio1-1* and *mta*, the *spa134* mutant exhibited relatively lower levels of mRNA methylation (Fig. 4l). Consistently, MeRIP analysis demonstrates that the *spa123* mutant is indeed impaired in the blue light-induced mRNA methylation (Extended Data Fig. 7b and Supplementary Table 7). The mRNA accessions that exhibit blue light-induced methylation in the WT but not mutants (m^6^A B/D non-hyper) show a 79% (1,106/1,407) overlap between the *spa123* and *cry1cry2* mutants or a 97% (1,373/1,407) overlap between the *spa123* and *fio1-1* mutants, respectively (Extended Data Fig. 7c,f). Similar to the *cry1cry2* mutant, the *spa123* mutant also showed an apparent decrease of m^6^A density at 3′ UTR of mRNAs in blue light (Extended Data Fig. 7d). The hypomethylated transcripts of light-grown *spa123* mutant show 38% (930/2,475) or 43% (1,057/2,475) overlap with the *cry1cry2* or *fio1-1* mutant, respectively (Extended Data Fig. 7e). These results are consistent with the partially overlapping but nonlinear functional relationships of CRYs, SPAs and FIO1. For example, CRYs regulate photoresponsive transcription and proteolysis in addition to mRNA methylation^1^, SPAs regulate photoresponsive proteolysis of many transcription factors^13^ in addition to mRNA methylation described in this report (Fig. 4l and Extended Data Fig. 7b), whereas FIO1 catalyses mRNA methylation^36,40–43^ that is a co-transcriptional process regulated by many factors^17,24^.

We then specifically examined photoresponsive mRNA methylation and translation of two *CHR* genes, *AT4G10300* and *AT4G39460* in the *spa* triple mutants, using the IP–quantitative polymerase chain reaction assay. As expected, the photoresponsive mRNA methylation of these *CHR* transcripts decreased significantly in not only the *cry1cry2* and *fio1-1* mutants, but also the *spa123* and *spa134* mutants grown in blue light (Fig. 4m,n). Importantly, the level of m^6^A methylation of these *CHR* transcripts remains unchanged in the *cry1cry2*, *fio1-1* or *spa123* and *spa134* mutants grown in darkness or the *mta* mutant grown in darkness or blue light (Fig. 4m,n). Moreover, the *AT4G10300* and *AT4G39460* transcripts showed significantly decreased photoresponsive translation in the *spa123* mutant (Extended Data Fig. 7h,i). These results are consistent with the hypothesis that explains how blue light may differentially regulate mRNA metabolism and chlorophyll homeostasis. According to this hypothesis, blue light induces LLPS and co-condensation of the CRY2/SPA1/FIO1 trimolecular complex to activate FIO1 within the condensates, resulting in the photoresponsive increase of m^6^A methylation and translation of the FIO1-specific mRNAs that encode the CHRs required to maintain chlorophyll homeostasis and photosynthesis in the light-grown plants (Fig. 4o).

## Discussion

In the present study, we discovered the function and mechanism of the METTL16-type m^6^A writer FIO1 in the control of chlorophyll homeostasis. We show that the CRY2/SPA1 complex undergoes light-induced LLPS to condense FIO1, resulting in activation of FIO1 and FIO1-specific m^6^A methylation and translation of mRNAs encoding at least six CHRs. Our results lead to new propositions with respect to how light regulates chlorophyll homeostasis. First, like CRYs and FIO1, *CHR* genes are not directly involved in chlorophyll synthesis or breakdown. The *CHR* genes share the CRY/FIO1-dependent light regulation of mRNA methylation and protein expression (Fig. 2i–m) and they share similar function of maintaining chlorophyll homeostasis without directly involved in chlorophyll metabolism (Supplementary Table 11 and references within). All except one CHR protein identified in this study are chloroplast proteins encoded by the nuclear genes. Those chloroplast CHR proteins are previously found to regulate chlorophyll homeostasis via various biochemical or cellular mechanisms, including β-cyanoalanine biosynthesis and cyanide detoxification (*AT3G61440* and *CYSC1*)^53^, regulation of photorespiration and osmotic stress responses (*AT4G10300* and *TRR14*)^49,62^, transportation of *S*-adenosylmethionine (SAM) to chloroplast (*AT4G39460* and *SAMT1*)^51^ and synthesis of phytohormones, such as auxin (*AT5G54810* and *TSB1*)^52,63,64^ and abscisic acid (*AT5G67030*, *ABA1* and zeaxanthin epoxidase)^65^. The only non-chloroplast CHR identified in this study (*AT1G01320* and *REC1*) is required for chloroplast compartmentation^54,66^, which may also affect chlorophyll homeostasis. It is conceivable that CHRs with the diverse biochemical functions would indirectly affect chlorophyll homeostasis by various mechanisms. For example, SAM is the cofactor of an CSE, Mg-protoporphyrin IX methyltransferase; TRR14 belongs to the cupin dioxygenase superfamily involved in catalysing a vast number of different biochemical reactions, REC1 controls chloroplast development and TSB1, ABA1 and CYSC1 may affect chlorophyll homeostasis via hormonal or stress responses. We identified the common mechanism regulating their mRNA metabolism in response to light, but exactly how CHRs and their associated biochemical reactions regulate chlorophyll homeostasis remains to be further investigated. Second, few of the six *CHR* genes showed a more than two-fold increase in light-induced m^6^A density, translation status or protein abundance in WT plants or a more than 50% decrease of these light responses in *cry1cry2* and *fio1-1* mutants (Fig. 2j–m). These results are consistent with the notion that ‘minor’ expression changes of multiple genes can collectively determine an important biological function, such as maintaining the appropriate chlorophyll homeostasis in response to light. Many biological functions of complex organisms, such as higher plants, are known to be determined by multiple genes, and these genes may each exert a ‘minor’ effect resulting from modest changes of gene expression in response to fluctuations of internal or external factors. Because our approach is based on the conventional omics analyses, the similar approach would be used to study other biological functions regulated by multiple genes with ‘minor’ expression changes. Third, genes encoding CSEs appear to be controlled by the photoregulatory mechanism distinct from that regulates CHRs. *Arabidopsis* genome encodes at least 58 CSEs, but none of them showed photoresponsive and genotype-specific changes in mRNA and protein expression or nuclear mRNA methylation and cytoplasmic translation that satisfactorily explain the low-chlorophyll phenotype of the *cry1cry2* and *fio1-1* mutants and the absence of the same phenotype in the *mta* mutant (Fig. 1a–c). For example, mRNAs of at least 12% *CSE* genes (7/58) exhibited light-induced increase of m^6^A methylation in WT plants (Supplementary Tables 12 and 13), which is about the average photoresponsive change in the epitranscriptome (2,399/27,655) (Extended Data Fig. 2a). However, none of these *CSE* mRNAs concomitantly showed corresponding changes of translation state as well as protein abundance to explain the low-chlorophyll phenotype in the *cry1cry2* and *fio1-1* mutants but not the *mta* mutant (Fig. 1). On the other hand, 48% of the *CSE* genes (28/58) showed light-induced increase of protein abundance in the WT seedlings, which is about 15-fold higher than the average photoresponsive changes of the proteome (895/27,655) (Supplementary Tables 1–13). This observation is consistent with the high demand of CSE proteins for chlorophyll synthesis and photosynthesis in light-grown plants. However, changes of the light promotion of *CSE* mRNA and protein expression of the *cry1cry2*, *fio1* and *mta* mutants detected in this study may explain only the phenotypes of individual mutants but not all three mutants at the same time (Supplementary Table 13). In contrast, the CHR hypothesis appears to satisfactorily explain the genotype-specific low-chlorophyll phenotype (Fig. 1c) by the mechanism that is consistent with the known biochemical activities of CRYs, FIO1 and MTA (Fig. 4o). Finally, it should be emphasized that, although our study does not show a direct role of MTA in light regulation of *CHRs* genes and chlorophyll homeostasis (Figs. 1c and 2), MTA does play important roles in light regulation of photosynthesis. For example, the key component of MTA complex, FKBP12 INTERACTING PROTEIN37 (FIP37), positively regulates photosystem PSI function in response to cold temperature^67^, whereas another protein of the MTA writer complex, VIRILIZER (VIR), positively regulates photoprotection and PSII function in response to high-light stress^20^. The phenotypic differences of different writer mutations probably result from different substrate specificities of individual m^6^A writers, but this proposition remains to be further investigated.

*Arabidopsis* CRYs interact and form LLPS with m^6^A writers MTA^15^, MOS4-associated complex subunits 3A and 3B (MAC3A/MAC3B)^68^ and FIO1 (Fig. 2) in the light-independent manner, which is in contrast to most CRY-interacting proteins reported so far^1^. We noticed that the transcriptome, m^6^A epitranscriptome, translatome and proteome changed in not only blue light-grown but also dark-grown *cry1cry2* mutant. In comparison with the WT seedlings, the numbers of genes (or m^6^A peaks) that exhibited statistically significant changes (FC <1/1.5 or >1.5, *P* < 0.05) are 7,417 (blue light) or 1,427 (dark) in transcriptome, 1,337 (peaks in blue light) or 479 (peaks in dark) in epitranscriptome, 9,774 (blue light) or 1,263 (dark) in translatome and 1,567 (blue light) or 174 (dark) in proteome (Extended Data Fig. 3). Although the overall changes of gene expression or RNA methylation in the dark-grown *cry1cry2* mutant are only 11–36% that of the light-grown *cry1cry2* mutant, the fact that the dark-grown *cry1cry2* mutant exhibited statistically significant changes in all four distinct omics datasets compared with that of the WT suggests that CRYs may have the light-independent or ‘dark’ functions. This phenomenon would be partially explained by the blue light-independent CRY2–writer interaction. The blue light-independent functions of CRYs have been previously reported^69,70^, and the light-independent activity has also been reported for other photoreceptors, such as phyA^71^. These results are consistent with a notion that photoreceptors are the photon-absorbing proteins that may have light-independent activity but change the activity upon absorption of photons.

We show in this study that CRYs mediate blue light-dependent LLPS of the *Arabidopsis* METTL16-type m^6^A writer FIO1 (Fig. 3). It has been previously reported that both *Arabidopsis* METTL3-type m^6^A writer MTA and the mammalian METTL3 are regulated by LLPS^15,72^, suggesting that LLPS is an evolutionarily conserved mechanism modulating the m^6^A writer activity. Our results shown in this report demonstrate that LLPS is the common mechanism underlying blue-light regulation of m^6^A writer activity and mRNA methylation in *Arabidopsis*. However, there are two distinct aspects of the CRY-mediated light regulation of MTA and FIO1. First, photoexcited CRY2 condenses MTA in the absence of other CRY2-signalling protein^15^, but photoexcited CRY2 condenses FIO1 in the SPA1-dependent manner (Fig. 3). Second, the light-induced condensation of the CRY2/SPA1/FIO1 complex is about ten times slower than the light-induced condensation of the CRY2/MTA complex (Fig. 3). CRY2 and SPA1 additively or synergistically activate FIO1 in vitro (Fig. 4), but it remains unclear whether CRY2 may directly activate MTA in vitro. Our results support a mechanistic model to explain how blue light differentially regulates FIO1 m^6^A writer and photomorphogenesis (Fig. 4o). According to this hypothesis, photoexcited CRY2 oligomerizes to increase its affinity to SPA1, forming the condensed nuclear CRY2/SPA1 photobody via light-induced LLPS. Over time, the condensed CRY2/SPA1 complex recruits and co-condenses FIO1 molecule to form the nuclear CRY2/SPA1/FIO1 co-condensate and increase the local concentration of these proteins (Fig. 4g), whereby the CRY2 and SPA1 additively or synergistically activate the m^6^A writer activity of FIO1 (Fig. 4h–j), resulting in sustained increase of m^6^A deposition at the RRACH-like sequences of mRNAs, and increased translation of these mRNAs. Many of these mRNAs encode CHR proteins that act to maintain the appropriate chlorophyll homeostasis in light-grown plants. The relatively slow kinetics of the CRY2/SPA1/FIO1 co-condensation is consistent with the sustained demands of *CHR*s for photosynthesis. Consistent with the hypothesis that the CRY2/SPA1 complex co-condenses FIO1 to stimulate its m^6^A writer activity, the SPA proteins are apparently required for the photoresponsive methylation and translation of the CHR transcripts. However, the exact biochemical mechanism underlying the CRY2/SPA1/FIO1 complex-mediated blue-light regulation of mRNA methylation and translation remains to be further investigated.

## Methods

### Plant materials and growth conditions

All WT, mutants and transgenic lines used in this study were in *Arabidopsis thaliana* Columbia (Col-4). *cry1cry2*, *CRY2–GFP*/*cry1cry2*, *CRY2–GFP*^*D387A*^/*cry1cry2*, *CRY2–GFP*^*P532L*^/*cry1cry2*, *35S::F–GFP*, *35S::SPA1–Myc ABI3::MTA/mta*, *fio1-1*, *spa123* and *spa134* have been described previously^15,36,60,61^, and *fio1-2* (SALK_209355) was ordered from ABRC. Transgenic populations were screened either on Murashige and Skoog agar medium containing 25 mg l^−1^ glufosinate (Cayman Chemical, catalogue number 16675) or on compound soil watered with BASTA solution. A light-emitting diode was used to generate monochromatic blue light (peak 450 nm; half-bandwidth of 20 nm), and cool white, fluorescent tubes were used for generating white light. The seedlings used in these experiments were grown in either a growth chamber (Conviron, model no. E7/2) or growth room at 21 °C under different light regimes.

### Protein expression constructs

To prepare *pACT2::Flag–FTO–GFP* plasmid, the coding sequence (CDS) of FIO1 was polymerase chain reaction-amplified. Mix the DNA fragments of FIO1 and XmaI-digested *pACT2::Flag–GFP* vector for in-fusion reaction (TaKaRa, catalogue number 639650). For *pFIO1::Flag–FIO1–GFP* or *pMTA::Flag–MTA–GFP* plasmids, the promoters were polymerase chain reaction-amplified from *Arabidopsis* genomic DNA and mixed with SacI/SpeI-digested *pACT2::Flag–FTO–GFP* or *pACT2::Flag–MTA–GFP* (*ACT2* promoter was removed) for in-fusion reaction.

To generate *35S::Flag–CRY2–YFP*, *35S::Flag–CRY2*^*D387A*^*–YFP*, *35S::FIO1–CFP*, *35S::Flag–SPA1–mCherry*, *35S::Flag–SPA1–BFP*, *35S::SPA1–HA*, *35S::Flag–H2B–mCherry* and *35S::Flag–H2B–BFP*, the CDS regions of genes were polymerase chain reaction-amplified using different templates. Each polymerase chain reaction fragment with HA, CFP, YFP, mCherry or BFP CDS was assembled into XmaI/BamHI-digested *35S::Flag–GFP* vector (GFP CDS was released) through in-fusion method.

For BiFC assays, the sequences encoding the N-terminal (nYFP; 1–157 amino acids) and C-terminal (cYFP; 158–239 amino acids) of YFP were amplified by polymerase chain reaction, which were mixed with *CRY2*, *CRY2*^*D387A*^, *CRY2*^*P532L*^, *MTA*, *FIO1*, *LUC*, *SPA1* or *SPA1*^*WD*^ CDS, respectively, for in-fusion into XmaI/BamHI-digested *35S::Flag–GFP* vector to produce *35S::CRY2–nYFP*, *35S::CRY2*^*D387A*^*–nYFP*, *35S::CRY2*^*P532L*^*–nYFP*, *35S::SPA1–nYFP*, *35S::SPA1*^*WD*^*–nYFP*, *35S::LUC–nYFP*, *35S::MTA–cYFP*, *35S::FIO1–cYFP* and *35S::LUC–cYFP*.

To generate *pQCMV–Flag–CRY2*, *pQCMV–Flag–CRY2*^*PHR*^ and *pQCMV–Flag–CRY2*^*CCE*^ for co-IP assays, polymerase chain reaction-amplified *CRY2*, *CRY2*^*PHR*^ and *CRY2*^*CCE*^ CDSs were in-fusion into SpeI/KpnI-digested *pQCMV–Flag–GFP* (GFP CDS was released). For preparing *pCMV–Myc–FIO1*, *pCMV–Myc–FIO1*^*MTD*^, *pCMV–Myc–FIO1*^*PCR*^ and *pCMV–Myc–mFIO1*, the CDSs of different versions of FIO1 were amplified by polymerase chain reaction and assembled into BamHI-digested *pCMV–Myc* vector by in-fusion. To create *pQCMV–Flag–CRY2–DsRED*, *pQCMV–Flag–MTA–YFP* and *pQCMV–Flag–FIO1–YFP* for microscopy, the polymerase chain reaction products of *CRY2*, *MTA* and *FIO1* were mixed with *YFP* or *DsRED* CDS for in-fusion into SpeI/KpnI-digested *pQCMV–Flag–GFP* vector.

For preparing *FIO1*, *FIO1*^*MTD*^, *FIO1*^*SAAG*^, *CRY2*^*PHR*^, *CRY2*^*CCE*^, *CRY2*^*CCE-P532L*^, *CRY1*^*PHR*^, *CRY1*^*CCE*^, *SPA1*, *SPA1*^*NKD*^ and *SPA1-WD* CDSs were amplified by polymerase chain reaction and assembled into *pGEX4-3* vector by in-fusion. For preparing *SPA1*, *SPA1*^*NKD*^, *SPA1-WD* and *SPA1*^*WD847*^ CDSs were amplified by polymerase chain reaction and assembled into *pET28a* vector by in-fusion. The primers used for plasmid constructions are listed in Supplementary Table 14. All cloned sequences in plasmids were validated by Sanger sequencing.

### Expression of proteins in HEK293T cells

HEK293T cells were grown in Dulbecco’s modified Eagle medium supplemented with 10% foetal bovine serum, 100 IU penicillin and 100 mg l^−1^ streptomycin at 37 °C and 5% CO_2_. About 2.4 × 10^6^ cells were seeded per 10-cm plate. For transfection, 10–15 μg of plasmid DNA was combined with 60 μl 2.5 M CaCl_2_ and diluted to 600 μl with ddH_2_O. A total of 600 μl of 2× HeBS (250 mM NaCl, 10 mM KCl, 1.5 mM Na_2_HPO_4_, 12 mM dextrose and 50 mM HEPES, pH 7.5, adjust the pH to 7.05) was added while vortexing. After 5 min, this mixture was applied to the cells. Subsequently, 6 ml of medium with 25 μM chloroquine was added. After 16–20 h, the medium was replaced. Cells were typically collected 36–48 h post-transfection.

### Immunoblot and co-IP assays

In co-IP experiments with HEK293T cells, cells were washed with phosphate-buffered saline and lysed in 1% Brij buffer (1% Brij-35, 50 mM Tris–HCl pH 8.0, 150 mM NaCl, 1 mM phenylmethylsulfonyl fluoride (PMSF) and 1× protease inhibitor cocktail). After centrifugation at 12,000*g* for 10 min at 4 °C, the supernatant was saved as ‘Input’ or incubated with 20 μl FLAG M2 beads (Sigma, catalogue number F2426) for 2 h at 4 °C (IP). Beads were washed five times with cold 1% Brij buffer. Proteins were eluted using 25 μl of 3× Flag peptide solution in 1% Brij buffer. Both ‘Input’ and ‘IP’ samples were mixed with 5× SDS (250 mM Tris–HCl pH 6.8, 10% SDS, 0.5 M dithiothreitol (DTT), 0.5% bromophenol blue and 50% glycerol) buffer and heated at 100 °C for 5 min. For co-IP in seedlings, tissues were ground in liquid N_2_ and homogenized in IP buffer (50 mM Tris–HCl pH7.4, 150 mM NaCl, 1% Triton X-100, 1 mM PMSF, 2 mM NaF and 1× protease inhibitor cocktail). Post-centrifugation at 14,000*g* for 20 min at 4 °C, supernatants were saved as ‘Input’ or incubated with GFP-trap beads for 2 h at 4 °C. Beads were washed four times with cold IP buffer and proteins eluted with 5× SDS buffer at 100 °C for 5 min. Samples were analysed on 10% SDS–polyacrylamide gel electrophoresis and transferred to nitrocellulose transfer membranes (Pall Corporation, catalogue number 66485). The primary antibodies are anti-CRY1 (1:3,000 dilution), anti-CRY2 (1:3,000 dilution)^73^, anti-Myc (1:5,000; Millipore) and anti-FLAG (1:3,000 dilution; Sigma, catalogue number F3165).

### Measurement of chlorophyll contents

The chlorophyll content was measured by the method described previously^74^. Fresh leaves were weighed, frozen in liquid N_2_ and ground to powder. Each sample was mixed with 10 ml cold 80% acetone and incubated overnight at 4 °C in the dark. After centrifuging at 10,000*g* for 15 min at 4 °C, 1 ml supernatant was measured for absorption at 646 nm and 663 nm against an 80% acetone blank. Chlorophyll concentrations were calculated using: chlorophyll *a* = 12.21 × *A*_663_ − 2.81 × *A*_646_ and chlorophyll *b* = 20.13 × *A*_646_ − 5.03 × *A*_663_.

### Protein expression and purification

*E. coli* BL21 codon plus (Agilent, catalogue number 230280) cells transformed with plasmids grew in Luria–Bertani medium at 37 °C until *A*_600_ of 0.8. Protein expression was induced with 0.3 mM isopropyl-β-d-thiogalactopyranoside, followed by 16-h growth at 18 °C. Post-collection, cells were lysed in phosphate-buffered saline and centrifuged to clear lysates, and recombinant proteins were isolated using glutathione-agarose resin (Pierce, catalogue number 16101) in a gravity-flow column. Recombinant proteins were eluted with the buffer (20 mM Tris, pH 8.0 containing 10 mM reduced glutathione). Eluates were concentrated with ultracentrifugal filters with the molecular mass cut-off of 10 or 50 kDa (Sigma, catalogue numbers UFC101096 and UFC501096), then desalted with Zeba Spin Desalting Columns (Thermo Fisher, catalogue number 89882), which was equilibrated with the buffer containing 20 mM Tris–HCl, pH 7.4 and 40% glycerol. The proteins were stored at −80 °C until use.

### In vitro methyltransferase activity measurement

The methyltransferase activity of FIO1 was determined with the MTase-Glo Methyltransferase Assay kit according to the manufacturer’s instructions (Promega, catalogue number V7602). For WT and mutated versions of FIO1 protein, 1 μM protein and 20 μM SAM were mixed in the reaction buffer containing 20 mM Tris pH 7.4. RNA substrates (Supplementary Table 15) were serially diluted to the concentration from 4 μM to 0 nM. The reactions were incubated for 30 min at 25 °C. Then 2 μl MTase-Glo Reagent (10×) was added to each reaction to convert SAH to ADP and 22 μl MTase-Glo Detection solution was added subsequently to transform ADP to ATP. The reactions were then transferred to a white 96-well microplate (Sigma, catalogue number CLS3603-48EA), and the luminescence was detected by Tecan Infinite F200. The luminescence value of the reaction without the RNA substrate was used to monitor the background, which was subtracted from the luminescence value of the reactions with the RNA substrate. Steady-state kinetics were determined by fitting the initial rates to the Michaelis–Menten equation using the GraphPad Prism 8.0 software.

### ELISA-based methyltransferase activity measurement

Reactions contained 5′-biotinylated RNA substrate (Supplementary Table 15) and 20 μM SAM in the reaction buffer (20 mM Tris pH 8.0). The reactions were initiated by adding 1 μM of FIO1 proteins and incubated at 25 °C for 30 min. To detect the production of methylated RNA, reactions were transferred to the 96-well neutravidin-coated plates (Pierce, catalogue number Pl15216) and incubated for 20 min at 4 °C. Followed by extensive washing and blocking, the plate was incubated first with a m^6^A-specific primary antibody (1:500 dilution, SYSY, catalogue number 202111), and subsequently with fluorescence-conjugated secondary antibody (1:1,000 dilution, Thermo Fisher Scientific, catalogue number A11369). m^6^A antibody binding was quantified by measuring the fluorescence at a wavelength of 790 nm (Li-COR). Reactions without SAM were used to measure the background due to non-specific binding of antibodies.

### Quantification of m^6^A level in RNA by LC–QQQ–MS/MS

m^6^A quantification by liquid chromatography–triple quadrupole–mass spectrometry/mass spectrometry (LC–QQQ–MS/MS) was performed as previously reported^75^. PolyA RNA was extracted from total RNA using the polyA-tail purification kit (Thermo Scientific, catalogue number 61012). In vitro RNA probes were ethanol isolated. These probes were digested with nuclease P1 (Sigma, catalogue number N8630) in a buffer (25 mM NaCl and 2.5 mM ZnCl_2_) at 42 °C for 1 h. FastAP Thermosensitive Alkaline Phosphatase (Thermo Fisher Scientific, catalogue number EF0651) was added and samples were incubated at 37 °C for 4 h. After filtering through a 0.22-mm filter (Millipore, catalogue number GSWP04700), samples were injected into an Agilent 6460 LC–MS/MS system. Nucleosides were identified by retention time and mass transitions (268 to 136 for A; 282 to 150 for m^6^A) and quantified against a standard curve from nucleoside standards.

### Image acquisition and analysis

Tobacco leaves transformed with indicated plasmids were incubated in the dark. Before observation, the tobacco leaves were transferred to the slides. The microscopic images were acquired using a Zeiss LSM 780 confocal microscope equipped with a Plan-Apochromat 40×/1.40 Oil DIC M27 objective. For BFP, mCherry or YFP signals, BFP was excited with 405 nm laser and detected at 450 nm, YFP was excited with 514 nm laser and detected at 520–620 nm and mCherry was excited with 561 nm laser and detected at 566–629 nm. For time-lapse imaging, a chamber (1 cm × 1 cm) was made on slides using SecureSeal adhesive sheets (120 µm in thickness; Grace Bio-Labs, catalogue number 620001). To observe CRY2 photobodies, the slide was put under a microscope and a 488 nm laser (2% of the laser power) was used to scan the samples. The images were captured in a time series with the first image captured with the 488 nm laser turned off (as T0) and the remaining images captured with the 488 nm laser on (1% of laser power). Image analysis was performed with FIJI/ImageJ^76^.

### FRAP assay

FRAP analysis of CRY2 photobodies or photobody-like complexes in cells was performed as reported before^15^. Photobodies were photobleached using laser pulses of 514 nm (100 iterations; 90% of laser power). Images of fluorescence recovery were captured every second for at least 1 min. The fluorescence intensities of both photobleached and non-photobleached areas in the photos were measured using FIJI/ImageJ to match the requirements of easyFRAP software version for further analysis^77^. Each fluorescence recovery curve was subjected to full-scale normalization to adjust for variations in pre-bleach intensity of photobleached areas, differences in total fluorescence and changes in bleaching depths across experiments. Then the normalized data were fitted with double exponential model: (*t*) = *I*_0_ − *α* × *e* − *βt* − *γ* × *e* − *δt* (where *I*_0_ is the summit or plateau of the curve; *α*, *β*, *γ* and *δ* are algorithm parameters defined by the EasyFRAP software for curve fitting). For the full-scale normalized curve with the maximum analytic time, mobile fraction equals *I*_0_. Mobility is defined as the recovery rate of fluorescence after photobleaching.

### Translatome analysis

One millilitre pulverized tissue was added to 5 ml polysome extraction buffer (PEB: 200 mM Tris, pH 9.0, 200 mM KCl, 25 mM egtazic acid, 35 mM MgCl_2_, 1% phosphotungstic acid ethanol, 1 mM DTT, 1 mM PMSF, 100 μg ml^−1^ cycloheximide and 50 μg ml^−1^ chloramphenicol) with 1% detergent mix (20% (w/v) polyoxyethylene, 20% (v/v) Triton X-100, 20% (v/v) octylphenyl-polyethylene glycol and 20% (v/v) polyoxyethylene sorbitan monolaurate 20) and incubated on ice. After homogenization, the mixture was rested on ice for 10 min and centrifuged at 16,000*g* for 15 min at 4 °C. The cleared supernatant was passed through Miracloth (Millipore) and 10% preserved for RNA isolation. Pre-washed anti-FLAG M2 protein beads (1.5 ml) were added and incubated at 4 °C for 2 h. Post incubation, beads were washed in washing buffer (200 mM Tris (pH 9.0), 200 mM KCl, 25 mM egtazic acid, 35 mM MgCl_2_, 1 mM DTT, 1 mM PMSF, 100 g ml^−1^ cycloheximide and 50 g ml^−1^ chloramphenicol) and resuspended with 300 μl washing buffer containing FLAG3 peptide (200 ng μl^−1^) and RNase inhibitor (Thermo Fisher, catalogue number N8080119). After a 30-min incubation at 4 °C, supernatant was collected post-centrifugation for RNA purification. The RNA was used for preparation of TRAP-seq libraries with TruSeq RNA Library Prep Kit (Illumina). The libraries from three biological repeats for each sample were sequenced on the Illumina HiSeq 2500 sequencing systems. Cleaned reads of TRAP-seq and input samples were aligned to the TAIR10 reference genome with Bowtie2 (v2.1.0)^78^. Translation efficiency abundance was measured by RNA-Seq by Expectation-Maximization using the default parameters^79^. The translation state was calculated by the formula (RPKM in TRAP-seq + 1)/(RPKM in input + 1). Differential translation analysis was conducted using edgeR^80^ with a threshold of *P* value <0.05 and FC >1.5 was used to determine whether there were any significant differences in translation between samples.

### RIP assay

Seedlings were collected and ground with liquid nitrogen and lysis with extraction buffer (10 mM Tris–HCl pH 7.5, 50 mM NaCl, 0.25% NP-40, 1 U μl^−1^ RNase inhibitor). Ten per cent of the extract was kept as input. The remainder was immunoprecipitated using GFP trap resin at 4 °C for 3 h and washed five times with extraction buffer. Total RNA was isolated (Zymo, catalogue number R2052) from the washed GFP trap resin or input extraction.

### m^6^A epitranscriptome analysis

Total RNA was isolated using Direct-zol RNA Miniprep Kits (Zymo, catalogue number R2052). MeRIP-seq was performed using the EpiMark *N*^6^-Methyladenosine Enrichment Kit (NEB, catalogue number E1610S). The libraries of two biological repeats for each sample were sequenced on Illumina Novaseq6000 instruments in pair-end mode with 100 bp per reads. The adapter sequence of m^6^A MeRIP raw reads was trimmed by Trim Galore^81^. The trimmed reads were aligned to the TAIR10 reference genome with Bowtie2 (v2.1.0)^78^ with the default settings. MeRIP track files in BigWig format were generated using bamCoverage of deepTools (v3.1.3) with RPKM normalization^82^ from de-duplicated reads of Samtools^83^. m^6^A peaks were called by MACS2 (v2.1.1) and annotated using ChIPseeker^84,85^. Differential peaks were called with a threshold of *P* value <0.05 and FC >1.5. m^6^A data metaplots were plotted by deepTools (v2.5.1)^86^.

### MeRIP–quantitative polymerase chain reaction and Ribo-tag reverse transcription quantitative polymerase chain reaction

Poly(A) RNA was purified from total RNA with two rounds of polyA-tail purification. m^6^A-IP with the purified poly(A) RNA was performed using the EpiMark *N*6-Methyladenosine Enrichment Kit. m^6^A and non-m^6^A spike-in RNA from this kit were used as the normalization controls for m^6^A level analysis in reverse transcription quantitative polymerase chain reaction. Relative changes were calculated using the ΔΔCt method.

RNA from TRAP was synthesized with oligo-dT primers using SuperScript IV First-Strand Synthesis System (Invitrogen, catalogue number 18091050). Quantitative polymerase chain reaction was performed with gene-specific primers and SYBR Green Quantitative Polymerase Chain Reaction SuperMix-UDG (Invitrogen, catalogue number 11733-038) on a Mx3005P Real-Time Polymerase Chain Reaction System (Stratagene). Translation state for tested genes was normalized to the input RNA. The related primers used are listed in Supplementary Table 14.

### Transcriptome analysis

Total RNA was used for preparation of RNA sequencing libraries with TruSeq RNA Library Prep Kit (Illumina, catalogue number RS-122-2001). The libraries from three biological repeats were sequenced on the Illumina HiSeq 2500 sequencing systems in pair-end mode with 150 bp per read. After sequencing, the pair-end reads were aligned to the *Arabidopsis* TAIR10 genome using Tophat-2.0.11 with anchor length longer than eight nucleotides for spliced alignments^87^. Only uniquely mapped reads were retained for subsequent analysis. The expression levels for gene models from TAIR10 were measured and normalized as fragments per kilobase of transcript per million mapped reads (FPKM)^88,89^.

### Proteome analysis

Two-hundred milligrams of fresh seedling powder was extracted with lysis buffer (1% sodium deoxycholate, 1% Triton X-100, 0.1% SDS, 150 mM NaCl, 50 mM Tris–HCl, pH 7.5 and 1× protease inhibitor), and 200 µg of protein was reduced with DTT and iodoacetamide. The digestion was carried out at 37 °C for 14 h using trypsin/lysine C mix. Protein digests were directly desalted via homemade C18 StageTips.

The instrument was LTQ Orbitrap Fusion Lumos mass spectrometer coupled with an easy nLC-1000 UPLC. Two micrograms of desalted and dried peptides was loaded at 2 μl min^−1^ onto the analytical column (omics high-resolution series monolithic capillary HPLC columns, 75 μM × 50 cm, Kyoto Monotech) and separated with a linear gradient of 120 min. The flow rate was controlled at 600 nl min^−1^, and the column temperature was kept at 50 °C. A linear gradient was applied for the peptide separation. It started with 5% mobile phase B (100% acetonitrile), raised to 8% phase B in 4 min, then increased to 20% phase B in 76 min. The percentage of phase B was later increased to 30% in 30 min, and finally reached 90% in another 2 min, and maintained at 90% for 8 min. The mass spectrometer was operated under data-independent acquisition mode. Key parameters were set as follows: 1, MS scan range 350–1,500 Da; resolution 120,000; automatic gain control (AGC) target 4 × 10^5^; maximum injection time 50 ms; 2, higher-energy collisional dissociation-MS/MS resolution 30,000; AGC target 2 × 10^5^; collision energy 32; and 3, HRMS1-data-independent acquisition (DIA) method was applied and three MS1 scans were interspersed with 20 DIA MS/MS variable windows (in total 60 DIA MS/MS scans).

Spectronaut default parameters (BGS Factory Settings (default)) were used to analyse the DIA raw data. Peptide retention times were automatically aligned according to the indexed retention time peptides. Precursor and protein thresholds were set as 1.0% and 5% FDR, respectively. Decoy database was generated by mutated strategy. The average peak area of the top three peptides with FDR less than 1.0% was used for protein quantification.

### Statistics and reproducibility

The independent experiments with similar results are shown in the figures. The western blots for Figs. 3a,b,d–g and Extended Data Fig. 4a–c were repeated twice, and those for Extended Data Figs. 1c,d and 4g were repeated once. The confocal images for Figs. 3h,m and 4g and Extended Data Figs. 5a–f and 6k were repeated five times, and those for Extended Data Fig. 4d–f were repeated three times.

### Reporting summary

Further information on research design is available in the Nature Portfolio Reporting Summary linked to this article.

## Supplementary information

Reporting Summary

Supplementary Tables 1–16 Supplementary Table 1. RNA sequencing analysis. Supplementary Table 2. All m^6^A-peaks. Supplementary Table 3. WT blue versus dark up peaks (2,555 peaks in 2,399 genes). Supplementary Table 4. *cry1cry2* blue versus dark up peaks (1,539 peaks in 1,489 genes). Supplementary Table 5. *fio1-1* blue versus dark up peaks (333 peaks in 309 genes). Supplementary Table 6. *mta* blue versus dark up peaks (798 peaks in 777 genes). Supplementary Table 7. *spa123* blue versus dark up peaks. Supplementary Table 8. Translatome dataset. Supplementary Table 9. Proteome datasets. Supplementary Table 10. Three types of genes. Supplementary Table 11. CHR genes. Supplementary Table 12. CSE genes. Supplementary Table 13. CSE datasets. Supplementary Table 14. Primers used. Supplementary Table 15. RNA substrates. Supplementary Table 16. The exact *P* values.

## Source data

Source Data Fig. 1 Unprocessed western blots for Fig. 3.

Source Data Fig. 2 Unprocessed western blots for Extended Data Fig. 1.

Source Data Fig. 3 Unprocessed western blots for Fig. 3 and Extended Data Figs. 1 and 4.

Source Data Extended Data Fig./Table 1 Statistical source data for Figs. 1–4 and Extended Data Figs. 1–7.

## Data Availability

The raw data of transcriptomes, m^6^A epitranscriptomes and translatomes reported in this paper are available at GEO database with accession numbers GSE226927 and GSE227150. The MS proteomics data have been deposited to the ProteomeXchange Consortium with the dataset identifier PXD040660.
