# Author Correction: Light-induced LLPS of the CRY2/SPA1/FIO1 complex regulating mRNA methylation and chlorophyll homeostasis in *Arabidopsis*

**DOI:** 10.1038/s41477-023-01613-8

**Published:** 2023-12-19

**Authors:** Bochen Jiang, Zhenhui Zhong, Lianfeng Gu, Xueyang Zhang, Jiangbo Wei, Chang Ye, Guifang Lin, Gaoping Qu, Xian Xiang, Chenjin Wen, Maureen Hummel, Julia Bailey-Serres, Qin Wang, Chuan He, Xu Wang, Chentao Lin

**Affiliations:** 1 Basic Forestry and Plant Proteomics Research Center, Fujian Agriculture and Forestry University, Fuzhou, China; 2 Department of Molecular Cell and Developmental Biology, University of California, Los Angeles, CA USA; 3 Department of Chemistry, The University of Chicago, Chicago, IL USA; 4 Center for Plant Cell Biology and Department of Botany and Plant Sciences, University of California, Riverside, CA USA; 5 Shandong Laboratory of Advanced Agricultural Sciences at Weifang, Peking University Institute of Advanced Agricultural Sciences, Weifang, China

**Keywords:** Cell signalling, Light responses

Correction to: *Nature Plants* 10.1038/s41477-023-01580-0, published online 8 December 2023.

In the version of the article initially published, the surname of Maureen Hummel was incorrect. The name has now been updated in the HTML and PDF versions of the article.

