# Supplementary figures and images for: Light-induced LLPS of the CRY2/SPA1/FIO1 complex regulating mRNA methylation and chlorophyll homeostasis in Arabidopsis
Source: Nat Plants. 2023 Dec 8;9(12):2042–58. doi: 10.1038/s41477-023-01580-0 (PMC10724061; doi:10.1038/s41477-023-01580-0)

Fig. 3a

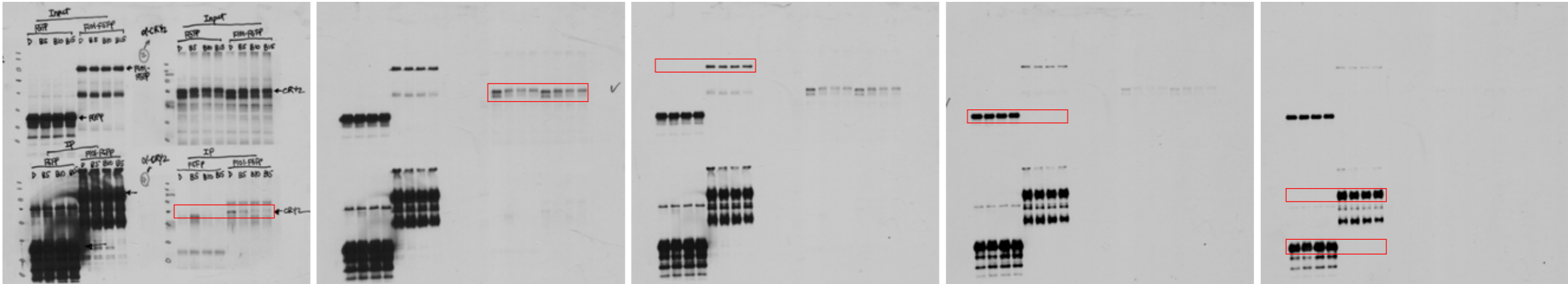

Fig. 3b

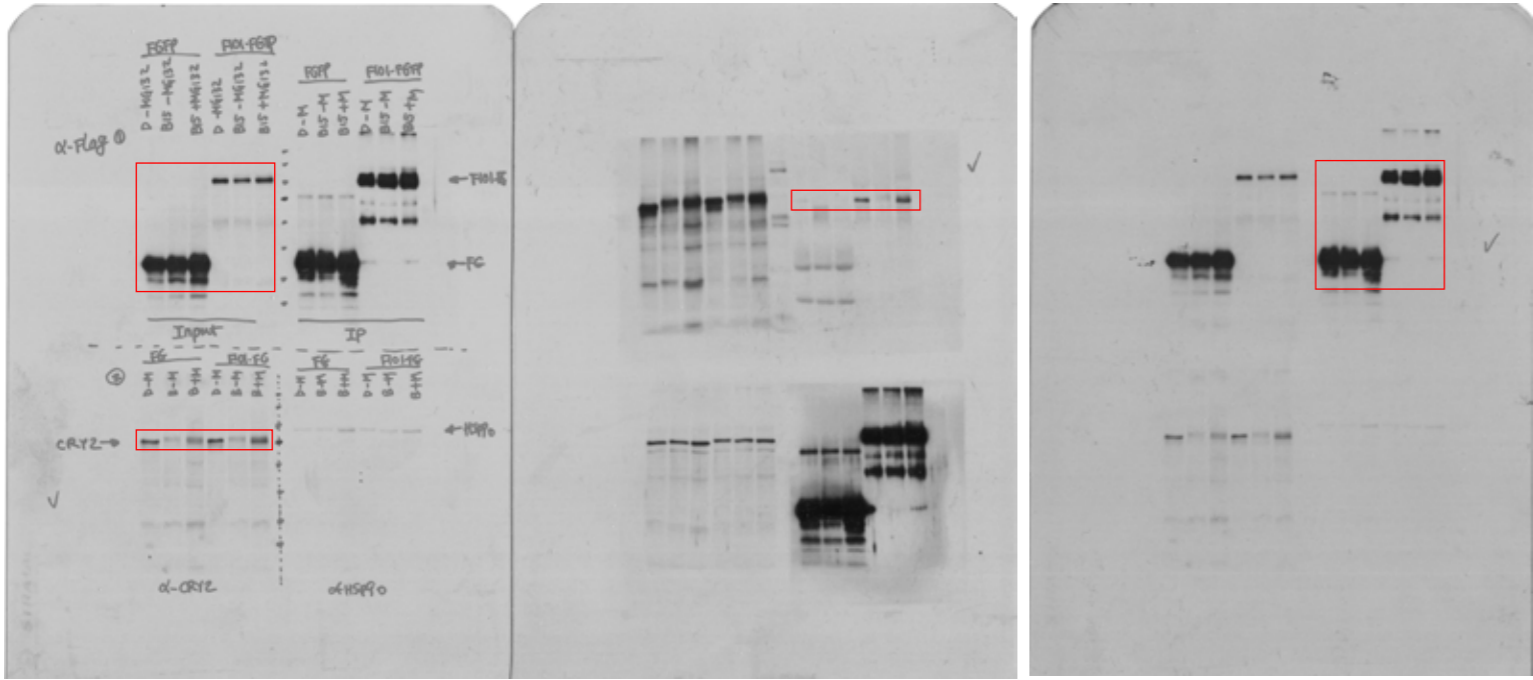

Fig. 3d

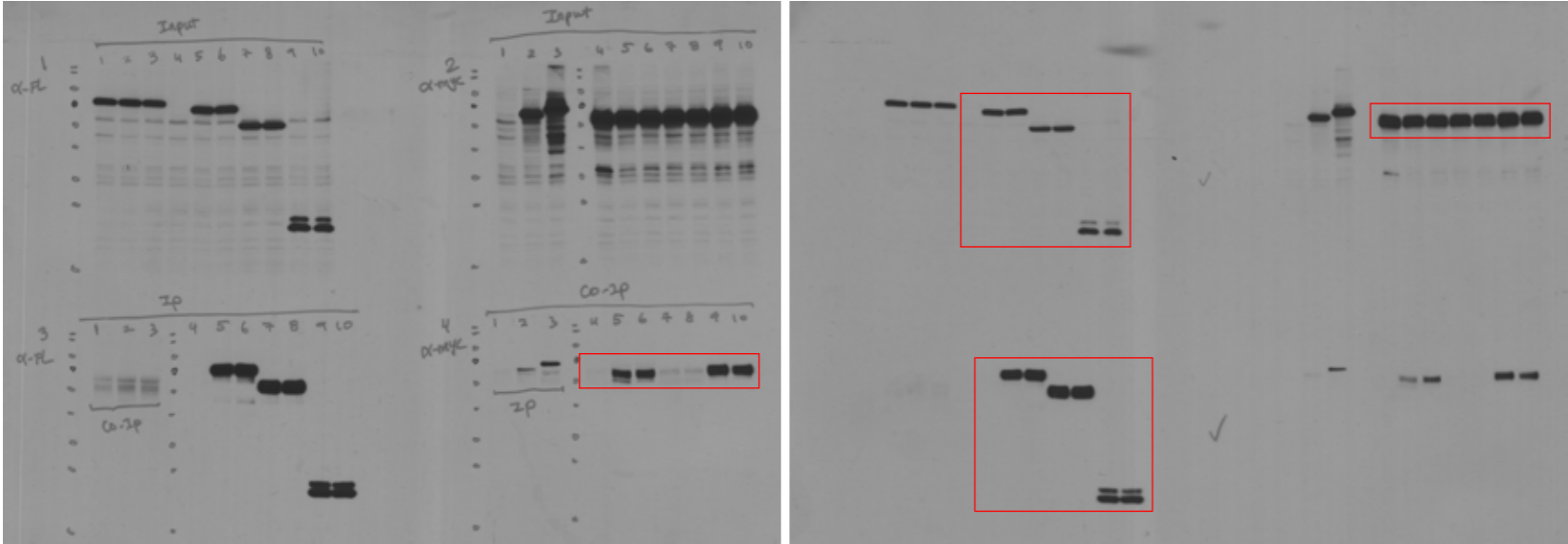

Fig. 3e

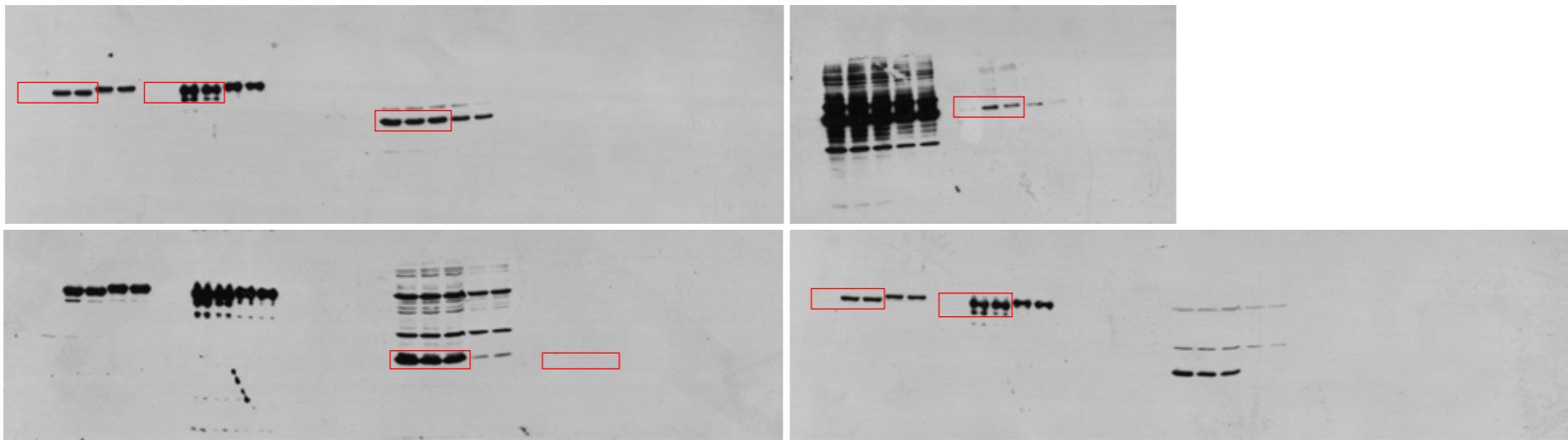

Fig. 3g

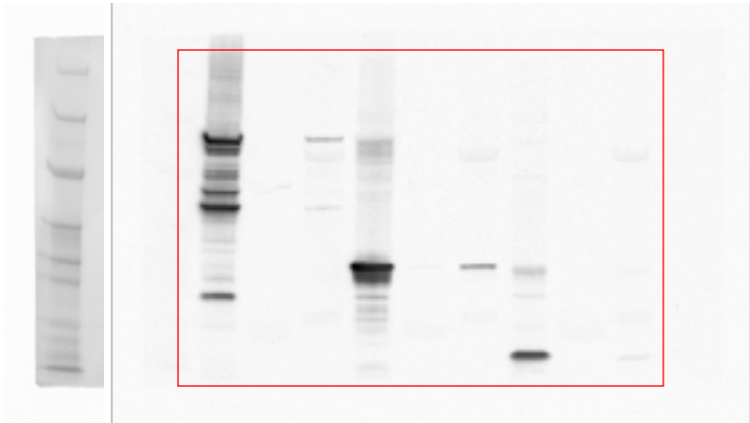

Supplement: Supplementary file 3 — Unprocessed western blots for Fig. 3. [file 41477_2023_1580_MOESM3_ESM.pdf]

Extended Fig.1c-d

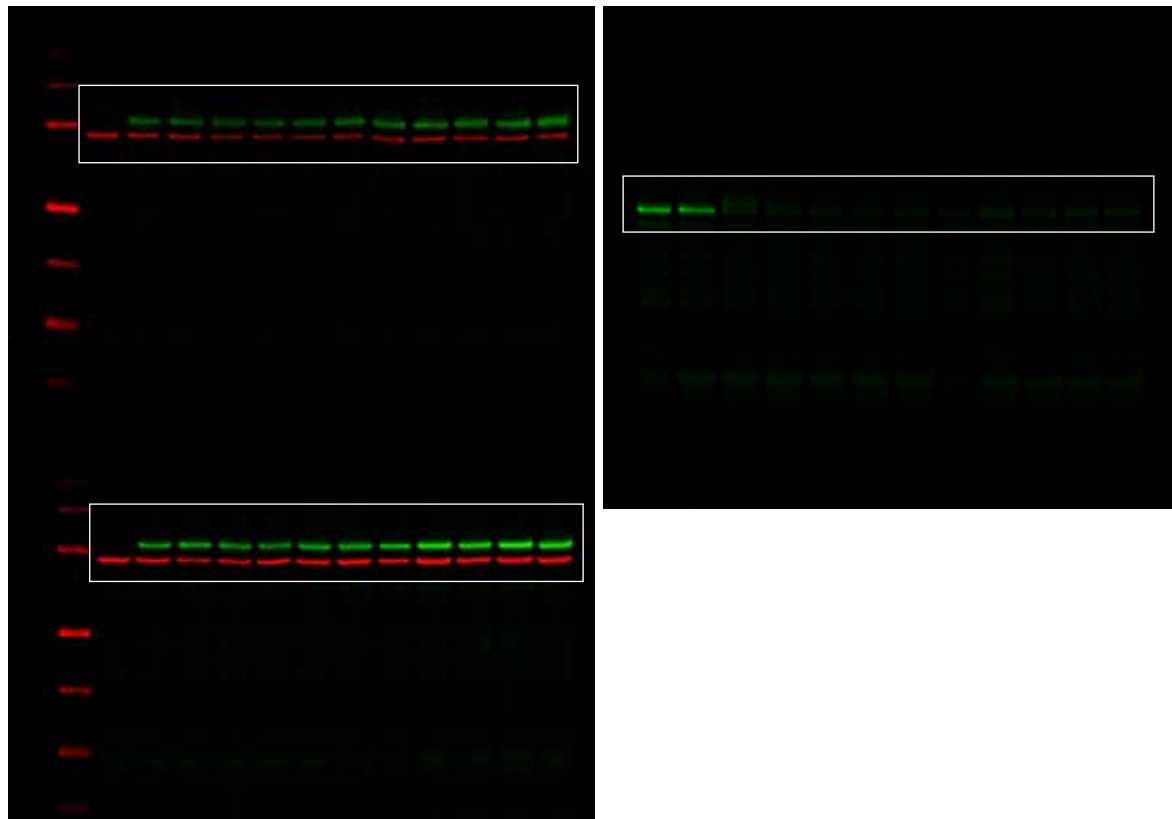

Extended Fig.1e

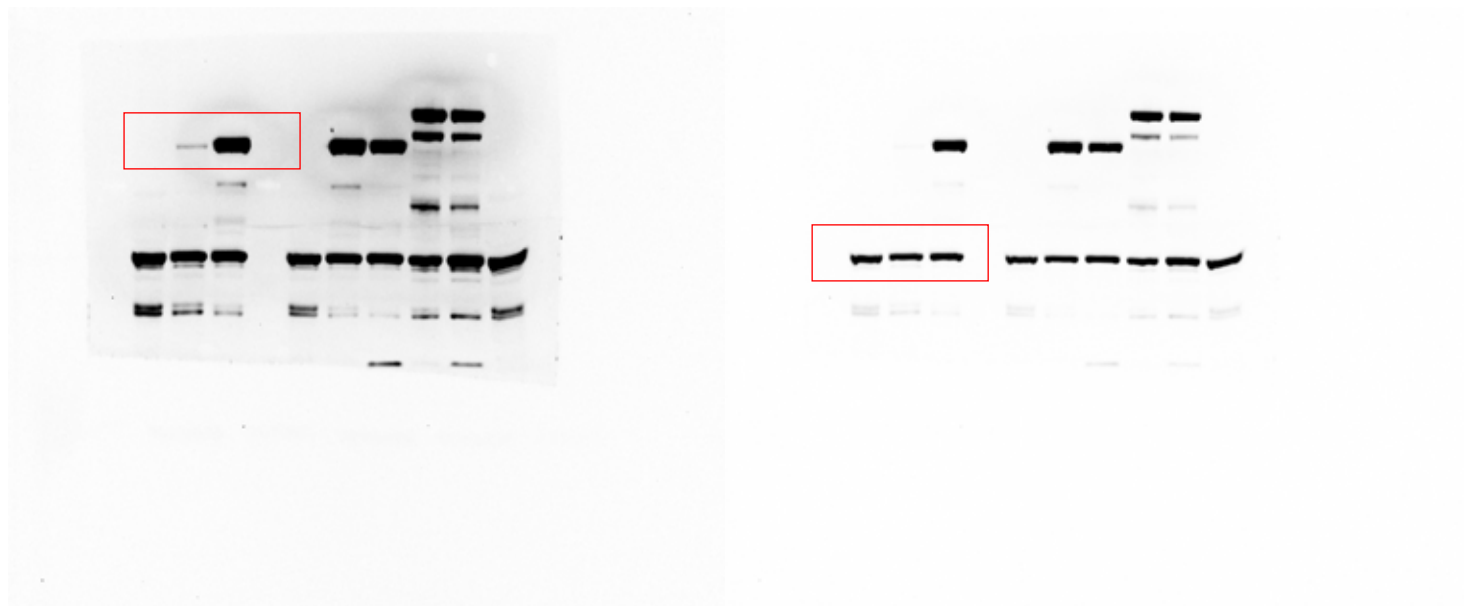

Supplement: Supplementary file 4 — Unprocessed western blots for Extended Data Fig. 1. [file 41477_2023_1580_MOESM4_ESM.pdf]

Extended Fig. 4a

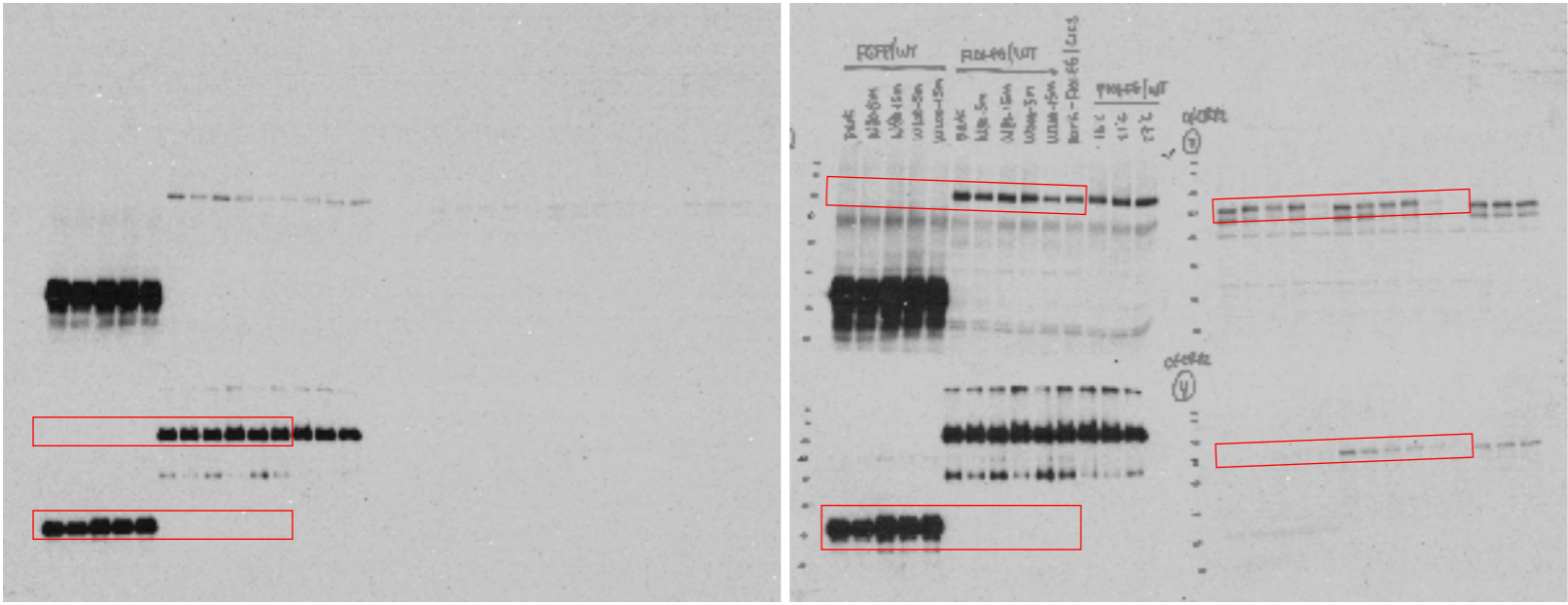

Extended Fig.4b

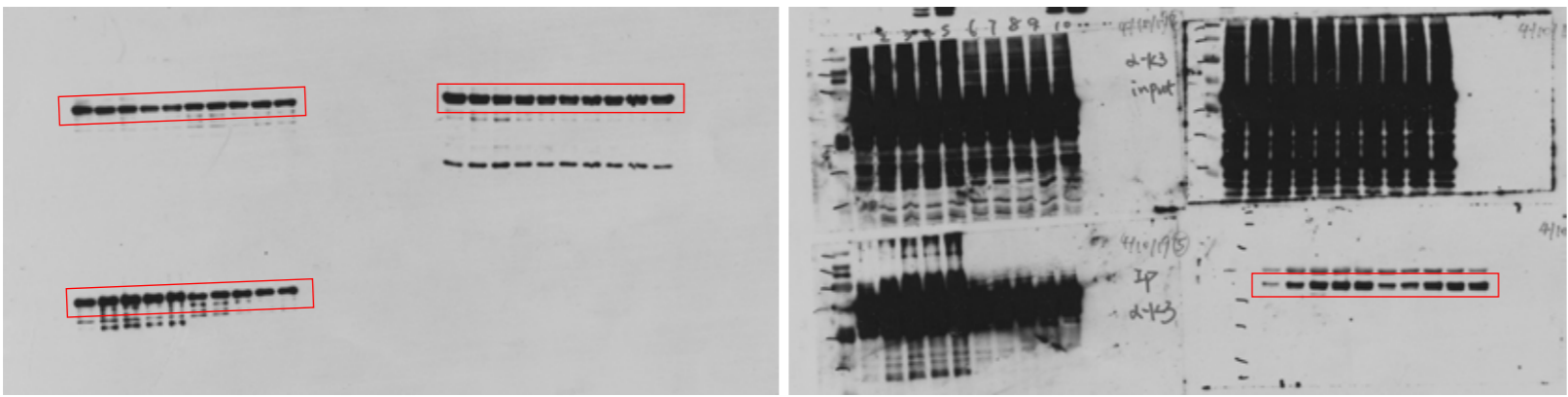

Extended Fig.4c

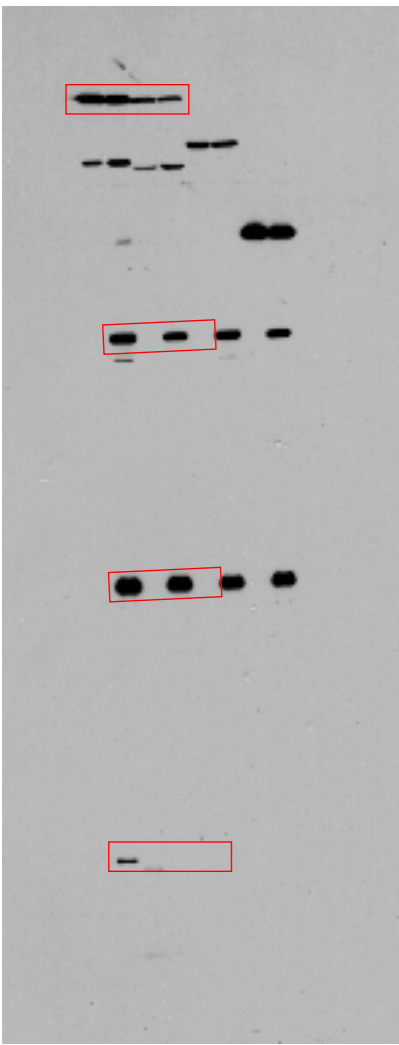

Extended Fig. 4g

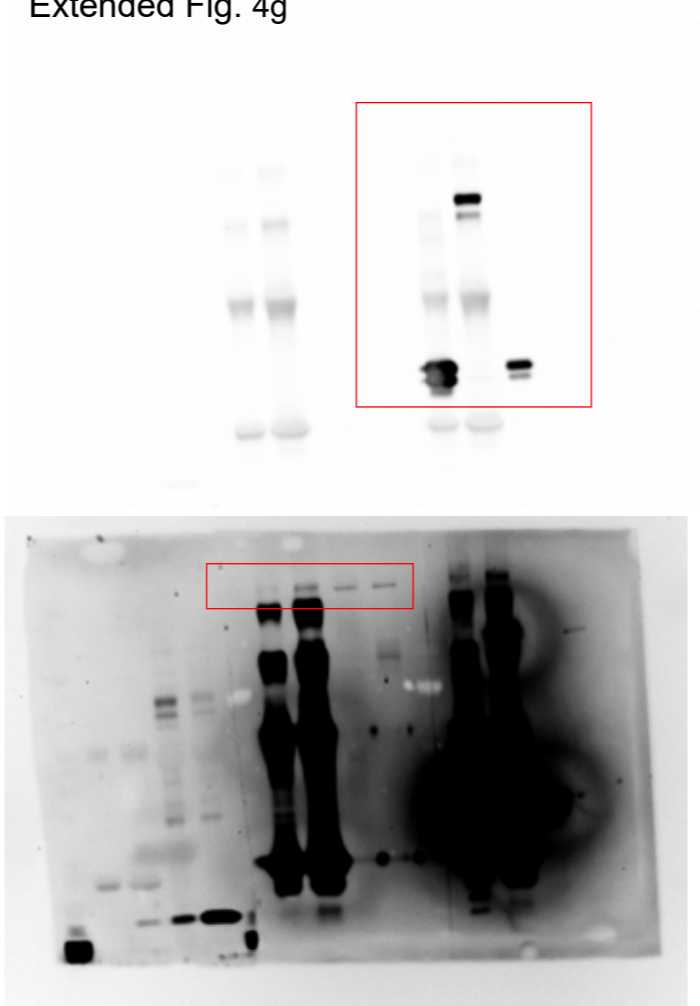

Supplement: Supplementary file 5 — Unprocessed western blots for Fig. 3 and Extended Data Figs. 1 and 4. [file 41477_2023_1580_MOESM5_ESM.pdf]
